# Modeling chaotic diabetes systems using fully recurrent neural networks enhanced by fractional-order learning

**DOI:** 10.1038/s41598-025-28637-8

**Published:** 2025-12-05

**Authors:** Esraa Mostafa, Tarek A. Mahmoud, Mohamed A. El-Brawany

**Affiliations:** Department of Industrial Electronics and Control Engineering, Faculty of Electronic Engineering, Menoufia University, Menouf, 32952 Egypt

**Keywords:** Fully recurrent neural network, Chaotic systems, Fractional order learning, Diabetes modeling, Computational biology and bioinformatics, Diseases, Engineering, Mathematics and computing

## Abstract

Modeling nonlinear medical systems plays a vital role in healthcare, especially in understanding complex diseases such as diabetes, which often exhibit nonlinear and chaotic behavior. Artificial neural networks (ANNs) have been widely utilized for system identification due to their powerful function approximation capabilities. This paper presents an approach for accurately modeling chaotic diabetes systems using a Fully Recurrent Neural Network (FRNN) enhanced by a Fractional-Order (FO) learning algorithm. The integration of FO learning improves the network’s modeling accuracy and convergence behavior. To ensure stability and adaptive learning, a Lyapunov-based mechanism is employed to derive online learning rates for tuning the model parameters. The proposed approach is applied to simulate the insulin-glucose regulatory system under different pathological conditions, including type 1 diabetes, type 2 diabetes, hyperinsulinemia, and hypoglycemia. Comparative studies are conducted with existing models such as FRNNs trained using gradient descent (FRNN-GD), Deep Feedforward Neural Network (DFNN, Diagonal RNNs with gradient descent (DRNN-GD), and DRNNs with FO learning (DRNN-FO). Simulation results confirm that the proposed FRNN-FO model outperforms these alternatives in terms of accuracy and robustness, making it a promising tool for modeling complex biomedical dynamics.

## Introduction

Modeling and analysis of chaotic systems are of great interest in chaos theory within the scientific community due to their inherent complexity, sensitivity to small changes in initial conditions, and the distinctive attractors that emerge when their phase states are plotted^1^. Moreover, these systems exhibit non-periodic behavior governed by fundamental physical laws, making them compelling subjects for both theoretical investigation and real-world applications^2^. Chaos theory has been successfully applied in diverse fields such as chemical reactions^3^, biology^4^, and medicine^5^. Consequently, there is a growing interest within the scientific community in understanding the nature and behavior of chaotic systems.

Chaotic system identification is a fundamental task in chaos theory. It involves estimating a black-box or grey-box model based on input - output data obtained from experimental observations, with or without prior knowledge of the underlying dynamics^6^. These models are essential for revealing system properties and predicting future behavior in response to specific inputs^7^. Accurate identification is also crucial for the control, analysis, and design of chaotic systems, motivating extensive research aimed at developing reliable and precise modeling approaches.

Artificial Neural Networks (ANNs) are widely used in machine learning and function approximation, particularly for estimating complex functions dependent on multiple inputs. Their popularity arises from key advantages such as self-adaptability, robustness, and the ability to adjust to dynamic environments, enabling them to track and respond to system changes effectively^8^. In the context of system identification, neural networks require only input-output data to approximate the unknown dynamics of nonlinear systems. Consequently, they are regarded as essential tools for data-driven system identification^9^. Significant progress has been made in developing various neural network architectures for identifying and characterizing chaotic systems^10,11^. Among these, recurrent neural networks (RNNs) have been extensively explored due to their ability to capture temporal dependencies and predict future states in chaotic dynamics. For instance, an Echo State Network was employed in^12^ to enhance large prediction horizons of chaotic time series. Similarly, the Recurrent Wavelet First-Order Neural Network has been successfully applied in several studies for chaotic system identification^10^.

Most medical systems exhibit complex nonlinear dynamics and have been shown to demonstrate chaotic behavior^13^, making them difficult to model using traditional approaches. Diabetes is a common disorder characterized by abnormally high blood sugar (glucose) levels due to insufficient insulin production or utilization^14^. It causes symptoms such as increased thirst and hunger and can lead to serious health complications, including heart disease, kidney failure, nerve damage, and vision problems^15^. Early identification of diabetes is essential to reduce long-term health risks. The glucose-insulin system is a highly complex biological process in which interactions between multiple components determine the system’s overall behavior. To better understand this relationship, researchers have proposed various models to simulate the dynamics between plasma glucose and insulin concentrations more accurately^16^. In these diabetes models, this relationship is inherently nonlinear and often influenced by the initial blood glucose concentration^17^. Given the effectiveness of chaos theory in analyzing complex dynamics, researchers have increasingly employed chaotic dynamics analysis to study physiological phenomena and improve the modeling of biological systems.

### Related works

To address the challenges posed by the nonlinear and chaotic nature of diabetes dynamics, researchers have widely applied neural networks for simulating and identifying diabetes-related processes. Several studies have investigated the use of neural network-based models for the identification of type 1 diabetes mellitus^18,19^. For example, a nonlinear model of type 1 diabetes was developed using a recurrent high-order neural network^20^. In parallel, machine learning techniques have been extensively explored for diabetes classification and prediction. For instance^21^, proposed a hybrid classification algorithm that combines supervised and unsupervised learning for predicting type 2 diabetes, while^22^ developed a highly accurate model using a deep neural network (DNN) trained through an unsupervised learning approach. Scalable implementations of machine learning algorithms in Hadoop-based clusters have also been employed for diabetes prediction^23^. Furthermore, deep learning methods were used in^24^ for classifying and forecasting type 2 diabetes mellitus.

### Motivation

Feed-forward and Recurrent Neural Networks are key architectures for modeling nonlinear systems, each with distinct strengths and applications. Recurrent Neural Networks (RNNs) are further divided into Fully Connected Recurrent Neural Networks (FRNNs) and Diagonal Recurrent Neural Network (DRNN)^25^. FRNNs can capture complex temporal dependencies and sequential patterns, making them well-suited for time-series prediction and dynamic system modeling. In contrast, DRNNs simplify the recurrence structure by incorporating self-feedback from the previous time step, thereby reducing computational complexity and memory requirements. The choice between FRNNs and DRNNs depends on the complexity of the problem and the trade-offs between modeling capacity and computational efficiency. The performance of RNNs also depends on the learning techniques applied during training. The performance of RNNs is also heavily influenced by the learning techniques used during training. The Backpropagation (BP) algorithm is widely adopted for parameter optimization; however, it often converges to local minimum in large-scale, nonlinear problems, limiting its effectiveness^26^. To address this, fractional calculus (FC) has been introduced to enhance neural network learning by improving convergence speed and accuracy. Integrating FC with BP has demonstrated superior performance compared to traditional integer-order methods^27^. This approach is particularly significant for modeling chaotic behaviors, such as those in the insulin-glucose system, where accurate representation is crucial for managing conditions like type 1 and type 2 diabetes, hypoglycemia, and hyperinsulinemia. Improved models enable more effective diagnostic tools and personalized treatments, potentially leading to better patient outcomes.

### Novelties and contributions

This paper develops a Fully Recurrent Neural Network (FRNN) identification model enhanced with a fractional-order (FO) learning algorithm, specifically designed for chaotic medical systems. To ensure convergence and stability, the model employs adaptive learning rates derived from Lyapunov stability theory. This integration enables the FRNN to effectively capture the complex dynamics of chaotic systems while maintaining robust learning stability. The study aims to validate the model’s ability to accurately predict the behavior of systems such as Diabetes Mellitus, highlighting the potential of FO-based RNNs in modeling chaotic biomedical phenomena. The main contributions of this paper are as follows:

1. Development of an identification approach using an FRNN integrated with a fractional-order learning algorithm (FRNN-FO), tailored for online training of network weights.
2. Stability assurance of the proposed model through the derivation of adaptive learning rates based on Lyapunov stability theory.
3. Performance evaluation of the proposed FRNN-FO framework by demonstrating its accuracy in predicting the dynamics of various diabetes models exhibiting chaotic behavior.

The remainder of this paper is organized as follows: Sect. 2 describes the chaotic behavior of the insulin-glucose regulatory system. In Sect. 3, we introduce the Fully Recurrent Neural Network (FRNN) based on the Fractional Order (FO) learning algorithm for modeling the chaotic medical system and discuss the convergence and stability analysis of the developed model. Section 4 presents simulation results for the identification of the various chaotic models of diabetes. Finally, conclusions are summarized in Sect. 5.

## Problem overview

 Diabetes mellitus is a chronic metabolic disorder characterized by abnormal blood glucose levels due to insufficient insulin production or improper insulin utilization^17^. The insulin-glucose regulatory system is highly nonlinear, and various mathematical models have been proposed to capture the dynamic interactions between insulin concentration, glucose concentration, and pancreatic β-cells under normal and pathological conditions^16^. A representative nonlinear model describing these interactions is given by:

$$\:{\dot{\text{x}}}_{1}=-{\text{w}}_{1}{\text{x}}_{1}+{\text{w}}_{2}{\text{x}}_{1}{\text{x}}_{2}+{\text{w}}_{3}{\text{x}}_{2}^{2}+{\text{w}}_{4}{\text{x}}_{2}^{3}+{\text{w}}_{5}{\text{x}}_{3}+{\text{w}}_{6}{\text{x}}_{3}^{2}+{\text{w}}_{7}{\text{x}}_{3}^{3}+{\text{w}}_{20}$$

$$\:{\dot{\text{x}}}_{2}=-{\text{w}}_{8}{\text{x}}_{1}{\text{x}}_{2}-{\text{w}}_{9}{\text{x}}_{1}^{2}-{\text{w}}_{10}{\text{x}}_{1}^{3}+{\text{w}}_{11}{\text{x}}_{2}\left(1-{\text{x}}_{2}\right)-{\text{w}}_{12}{\text{x}}_{3}-{\text{w}}_{13}{\text{x}}_{3}^{2}-{\text{w}}_{14}{\text{x}}_{3}^{3}+{\text{w}}_{21}$$

1 $$\:{\dot{\text{x}}}_{3}={\text{w}}_{15}{\text{x}}_{2}+{\text{w}}_{16}{\text{x}}_{2}^{2}+{\text{w}}_{17}{\text{x}}_{2}^{3}-{\text{w}}_{18}{\text{x}}_{3}-{\text{w}}_{19}{\text{x}}_{2}{\text{x}}_{3}$$

In this model, the state variables $\:{\text{x}}_{1},{\text{x}}_{2}$ and $\:{\text{x}}_{3}$ ​ represent insulin, glucose, and β-cell concentrations, respectively. Table 1 summarizes the physiological significance, nominal values, and corresponding units of the system parameters used in this study to model the glucose-insulin regulatory dynamics.

**Table 1:** Parameters of the Insulin-Glucose regulatory System.

Parameter	Physiological significance	Nominal values
$\:{w}_{1}$	Natural reduction of insulin concentration in the absence of glucose	2.04
$\:{w}_{2}$	Propagation rate of insulin in response to glucose presence	0.1
$\:{w}_{3}$	Rate of insulin secretion in response to glucose increase	1.09
$\:{w}_{4}$	Insulin secretion rate by β-cells, independent of other components	-1.08
$\:{w}_{5}$	Effect of insulin on glucose metabolism	0.03
$\:{w}_{6}$	Rate of glucose reduction due to insulin secretion	-0.06
$\:{w}_{7}$	Natural glucose production in the absence of insulin	2.01
$\:{w}_{8}$	Rate of glucose reduction due to β-cell-secreted insulin	0.22
$\:{w}_{9}$	Rate of β-cell proliferation due to increased glucose concentration	-3.84
$\:{w}_{10}$	Rate of β-cell decay due to its current level	-1.2
$\:{w}_{11}$	Rate of insulin secretion in response to glucose increase	0.3
$\:{w}_{12}$	Insulin secretion rate by β-cells, independent of other components	1.37
$\:{w}_{13}$	Effect of insulin on glucose metabolism	-0.3
$\:{w}_{14}$	Rate of glucose reduction due to insulin secretion	0.22
$\:{w}_{15}$	Natural glucose production in the absence of insulin	0.3
$\:{w}_{16}$	Rate of glucose reduction due to β-cell-secreted insulin	-1.35
$\:{w}_{17}$	Rate of β-cell proliferation due to increased glucose concentration	0.5
$\:{w}_{18}$	Rate of β-cell decay due to its current level	-0.42
$\:{w}_{19}$	Rate of insulin secretion in response to glucose increase	-0.15
$\:{w}_{20}$	Base rate of insulin secretion	-0.19
$\:{w}_{21}$	Base rate of glucose production	-0.56

This study investigates the chaotic behavior of the insulin-glucose regulatory system by analyzing its response to varying model parameters. By adjusting specific model parameters, various pathological conditions of diabetes can be simulated, illustrating the system’s transition from stability to chaos. The primary disorders of the insulin-glucose regulatory system that exhibit chaotic behavior under certain parameter variations are described as follows^28^:

### Type 2 diabetes

Characterized by insulin resistance and elevated glucose levels, this condition is modeled by reducing $\:{\text{w}}_{8}\:$​, which reflects a decreased effect of insulin on glucose uptake, leading to chaotic glucose fluctuations.

### Hypoglycemia

Hypoglycemia occurs due to excessive insulin accumulation in the bloodstream^10^. When the natural insulin decay rate, represented by $\:{\text{w}}_{1}$, is reduced, the system exhibits chaotic dynamics, signifying abnormal glucose depletion.

### Hyperinsulinemia

 Hyperinsulinemia is characterized by excessive insulin secretion from β-cells in response to prolonged high glucose levels^13^. In the model, increasing.

(insulin secretion rate by β-cells) destabilizes the system, leading to chaotic fluctuations in insulin levels.

### Type 1 diabetes

Type 1 diabetes results from the autoimmune destruction of β-cells, impairing insulin production. The model reflects this condition when the β-cell proliferation rate, represented by $\:{\text{w}}_{15}$​, diminishes. At low $\:{\text{w}}_{15}$ values, the system enters a chaotic regime, preventing proper glucose regulation.

The nonlinear and chaotic nature of diabetes dynamics demands advanced modeling techniques for accurate prediction and control. By enhancing FRNNs with a fractional-order (FO) learning algorithm, this research aims to improve the insulin-glucose regulatory system identification, forecasting, and ultimately the early diagnosis and optimization of treatment strategies, thereby deepening our understanding of diabetes progression.

## FRNN based on the fractional order (FO) learning algorithm

Recurrent Neural Networks (RNNs) are used to model time-dependent data by using feedback connections within their hidden layers. The structure of these networks can vary, with Diagonal RNNs (DRNNs) and Fully Recurrent RNNs (FRNNs) being two common types^25^. This section explains both DRNN and FRNN structures, introduces a fractional-order learning method for FRNN, and discusses the stability and convergence of the proposed model for chaotic diabetes system identification.

### Structure of DRNN

**Fig. 1:**
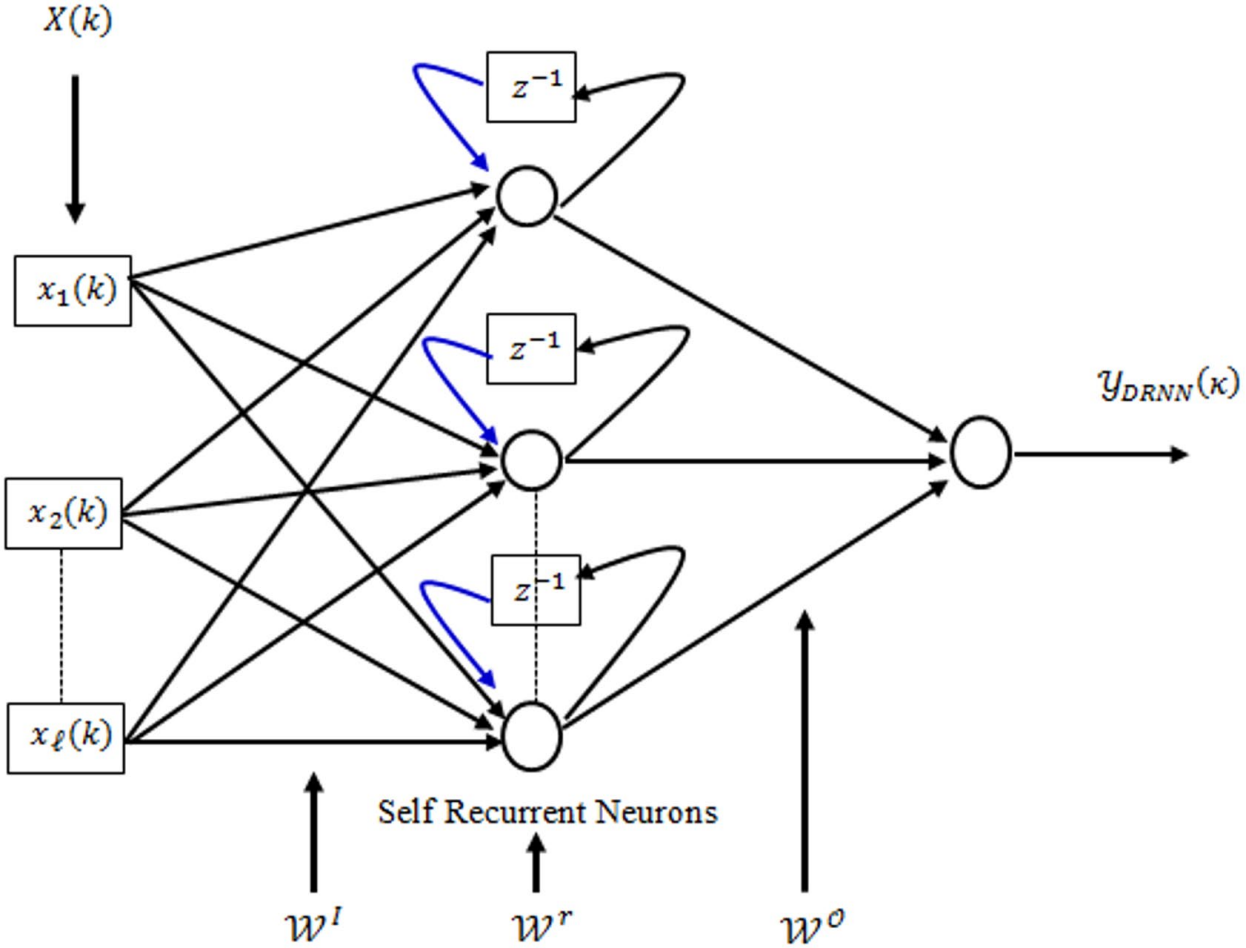
Structure of DRNN.

The DRNN shown in Fig. 1 has three layers. Each hidden neuron has a self-feedback loop, receiving its own previous output and the weighted sum of current inputs from the input layer. The hidden layer output is:

2 $$\:{\mathcal{Y}}_{\mathcal{m}}^{\mathcal{H}}\left({\upkappa\:}\right)=\mathcal{F}\left({\text{n}\text{e}\text{t}}_{\mathcal{m}}^{\mathcal{H}}\left({\upkappa\:}\right)\right)$$

where $\:\mathcal{F}$ is the nonlinear activation function. The induced field for any mth recurrent neuron is computed as:

3 $$\:{{\text{n}\text{e}\text{t}}_{\mathcal{m}}^{\mathcal{H}}\left({\upkappa\:}\right)={\mathcal{W}}_{\text{m}}^{\text{r}}\mathcal{Y}}_{\mathcal{m}}^{\mathcal{H}}\left({\upkappa\:}-1\right)+\sum\:_{\mathcal{l}}^{\text{L}}{\mathcal{W}}_{\text{m}\mathcal{l}}^{\text{I}}{{\upchi\:}}_{\mathcal{l}}$$

Here, $\:{{\upchi\:}}_{\mathcal{l}}\:$is the input vector, $\:{\mathcal{W}}_{\mathcal{m}}^{\text{I}}$ the input weight, and $\:{\mathcal{W}}_{\mathcal{m}}^{\text{r}}$ the self-feedback weight. The DRNN output uses a linear activation function:

4 $$\:{\mathcal{Y}}_{\text{D}\text{R}\text{N}\text{N}}\left({\upkappa\:}\right)=\sum\:_{\mathcal{m}=1}^{\mathcal{M}}{\mathcal{W}}_{\mathcal{m}}^{\mathcal{O}}{\mathcal{Y}}_{\mathcal{m}}^{\mathcal{H}}\left({\upkappa\:}\right)$$

where $\:{\mathcal{W}}^{\mathcal{O}}$ represents the output weight vector connecting the hidden layer neurons to the output neuron.

### Structure of FRNN

**Fig. 2:**
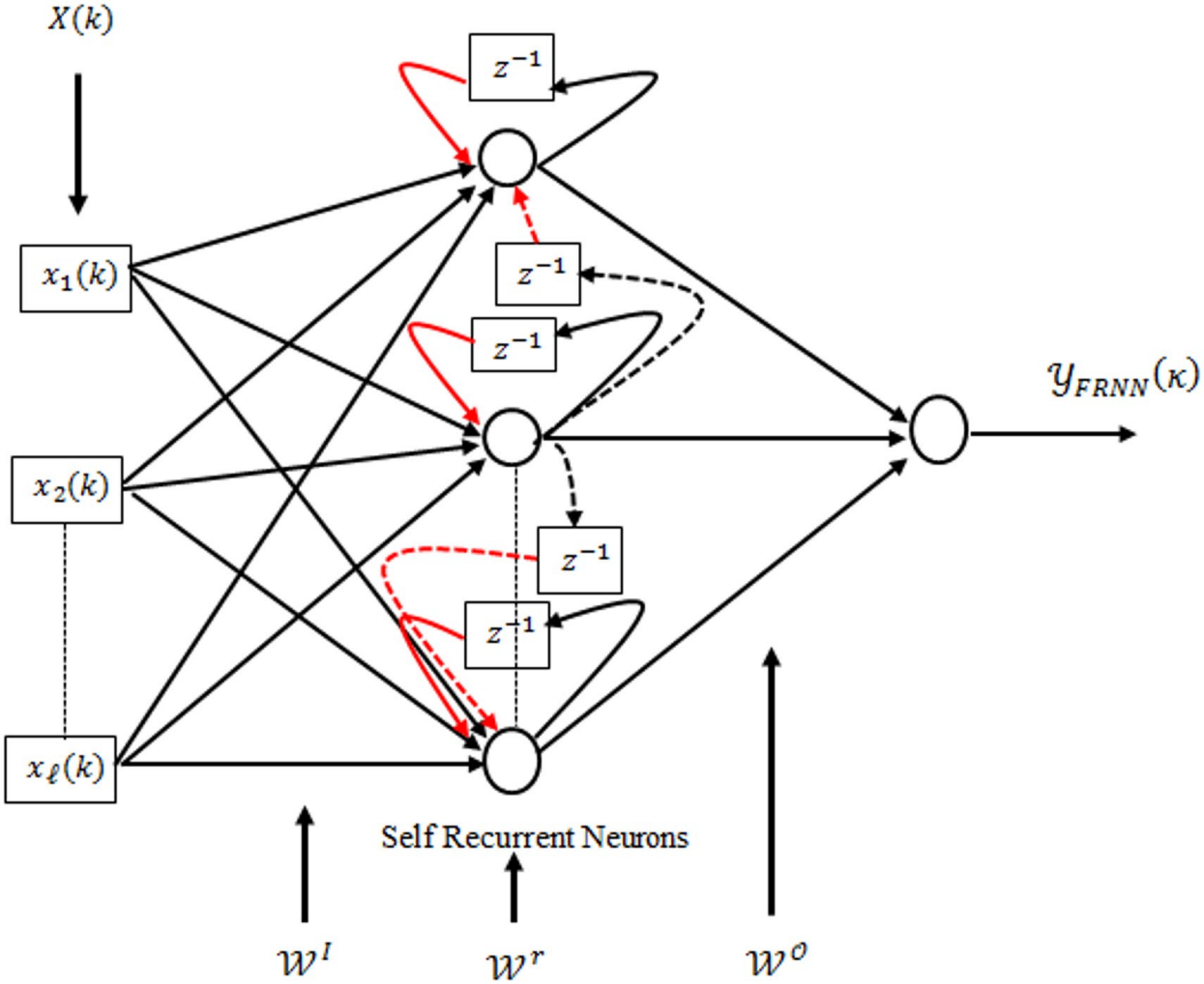
Structure of fully recurrent neural network.

The FRNN in Fig. 2 has a more complex structure than the DRNN, with each hidden neuron receiving both self-feedback and feedback from other neurons. The external input vector, $\:{{\upchi\:}}_{\mathcal{l}}$ is processed through the input layer with corresponding weights$\:\:{\mathcal{W}}^{\text{I}}$. The hidden layer output is determined as:

5 $$\:{\mathcal{Y}}_{\mathcal{m}}^{\mathcal{H}}\left({\upkappa\:}\right)=\mathcal{F}\left({\text{n}\text{e}\text{t}}_{\mathcal{m}}^{\mathcal{H}}\left({\upkappa\:}\right)\right)$$

where the induced field is calculated by:

6 $$\:{\text{n}\text{e}\text{t}}_{\mathcal{m}}^{\mathcal{H}}\left({\upkappa\:}\right)=\sum\:_{\mathcal{l}}^{\text{L}}{\mathcal{W}}_{\text{m}\mathcal{l}}^{\text{I}}{{\upchi\:}}_{\mathcal{l}}+\sum\:_{\text{j}=1}^{\mathcal{M}}{{\mathcal{W}}_{\text{m}\text{j}}^{\text{r}}\mathcal{Y}}_{\mathcal{m}}^{\mathcal{H}}\left({\upkappa\:}-1\right)$$

$\:{\mathcal{W}}_{\mathcal{m}}^{\text{I}}$ is the weight connecting the l^th^ input to the m^th^ hidden neuron and $\:{\mathcal{W}}_{\mathcal{m}}^{\text{r}}$ represents the recurrent weight connecting the j^th^ hidden neuron to the m^th^ hidden neuron. FRNN uses a linear activation function like DRNN.

7 $$\:{\mathcal{Y}}_{\text{F}\text{R}\text{N}\text{N}}\left({\upkappa\:}\right)=\sum\:_{\mathcal{m}=1}^{\mathcal{M}}{\mathcal{W}}_{\mathcal{m}}^{\mathcal{O}}{\mathcal{Y}}_{\mathcal{m}}^{\mathcal{H}}\left({\upkappa\:}\right)$$

where $\:{\mathcal{W}}^{\mathcal{O}}$represents the output weight vector connecting the hidden layer to the output neuron.

To enhance the effectiveness of system identification and improve the predictive capability of the FRNN model, a specialized learning algorithm must be employed. The next section introduces a Fractional-Order Learning Algorithm, which is designed to optimize the parameters of the FRNN in an efficient and adaptive manner.

### Fractional-order learning algorithm for FRNN model

An efficient fractional-order (FO) learning algorithm is developed in this section to optimize the parameters of the FRNN structure shown in Fig. 2. The primary objective of this algorithm is to determine the optimal weights that minimize a predefined cost function, given by:

8 $$\:\mathbb{E}\left({\upkappa\:}\right)=\frac{1}{2}{\left[\text{e}\left({\upkappa\:}\right)\right]}^{2}=\frac{1}{2}{\left[\mathcal{Y}\left({\upkappa\:}\right)-{\mathcal{Y}}_{\text{F}\text{R}\text{N}\text{N}}\left({\upkappa\:}\right)\right]}^{2}$$

Where $\:\text{e}\left({\upkappa\:}\right)$ denotes the instantaneous modeling error defined as the difference between the actual system output $\:\mathcal{Y}\left({\upkappa\:}\right)$ and FRNN model output $\:{\mathcal{Y}}_{\text{F}\text{R}\text{N}\text{N}}\left({\upkappa\:}\right)$ .

The FO learning algorithm utilizes the gradient descent method to iteratively update the network parameters, following the rule:

9 $$\:\frac{\text{d}{\uptheta\:}}{\:\text{d}\text{t}}=-{{\upeta\:}}_{{\uptheta\:}}\frac{\partial\:\mathbb{E}}{\partial\:{\uptheta\:}}$$

Where $\:{\uptheta\:}=\left[{\mathcal{W}}_{\text{m}\mathcal{l}}^{\text{I}}\:\:{\mathcal{W}}_{\text{m}\text{j}\:\:}^{\text{r}}{\mathcal{W}}_{\mathcal{m}}^{\mathcal{O}}\right]$ is the vector of adjustable weights, including input, recurrent, and output weights of the FRNN model. The parameter $\:{{\upeta\:}}_{{\uptheta\:}}\:$represents the learning rate.

A key advantage of fractional calculus (FC) is its ability to incorporate the influence of past information on current variables, leading to more accurate results. This research employs the Grunwald-Letnikov (GL) definition for fractional-order derivatives, given by:

10 $$\:{}_{\text{a}}{}^{\:}{\text{D}}_{\text{t}}^{{\upalpha\:}}\mathcal{x}\left(\text{t}\right)=\text{f}\left(\text{t},\mathcal{x}\left(\text{t}\right)\right)=\underset{\mathcal{h}\to\:0}{\text{lim}}\frac{1}{{\mathcal{h}}^{{\upalpha\:}}}{\sum\:}_{{\upkappa\:}=0}^{\left[\frac{\text{t}-\mathcal{b}}{\mathcal{h}}\right]}{\left(-1\right)}^{{\upkappa\:}}\left(\begin{array}{c}\alpha\:\\\:\kappa\:\end{array}\right)\mathcal{x}\left[\text{t}-{\upkappa\:}\mathcal{h}\right]$$

where the binomial coefficient is defined as:

$$\:\left(\begin{array}{c}\alpha\:\\\:\kappa\:\end{array}\right)=\frac{{\Gamma\:}({\upalpha\:}+1)}{{\upkappa\:}!{\Gamma\:}({\upalpha\:}-{\upkappa\:}+1)}$$

The Gamma function $\:{\Gamma\:}$ used in the above equation can be defined by the following equation:

11 $$\:{\Gamma\:}\left({\upalpha\:}\right)=\underset{0}{\overset{{\infty\:}}{\int\:}}{\text{t}}^{{\upalpha\:}-1}\:{\text{e}}^{\text{t}}\:\text{d}\text{t}$$

Using the GL fractional-order derivative in the gradient descent learning rule, the weight update equation is expressed as:

12 $$\:{}_{\text{a}}{}^{\:}{\text{D}}_{\text{t}}^{{\upalpha\:}}{\uptheta\:}\left({\text{t}}_{{\upkappa\:}}\right)=\text{f}\left({\text{t}}_{{\upkappa\:}},{\uptheta\:}\left({\text{t}}_{{\upkappa\:}}\right)\right)=-{{\upeta\:}}_{{\uptheta\:}}\frac{\partial\:\mathbb{E}}{\partial\:{\uptheta\:}}$$

Applying the numerical solution for the fractional-order equation, the weight update rule for the FRNN parameters is obtained as:

13 $$\:{\uptheta\:}\left({\upkappa\:}+1\right)=-{{\upeta\:}}_{{\uptheta\:}}\frac{\partial\:\mathbb{E}\left({\upkappa\:}\right)}{\partial\:{\uptheta\:}\left({\upkappa\:}\right)}{\mathcal{h}}^{{\upalpha\:}}-{\sum\:}_{\mathcal{j}=0}^{{\upkappa\:}}{\text{C}}_{\mathcal{j}}^{{\upalpha\:}}{\uptheta\:}\left({\upkappa\:}\right)$$

where $\:{\text{C}}_{\mathcal{j}}^{{\upalpha\:}}$ is recursively defined as:

14 $$\:{\text{C}}_{\mathcal{j}}^{{\upalpha\:}}=\left\{\begin{array}{c}1\:\:\:\:\:\:\:\:\:\:\:\:\:\:\:\:\:\:\:\:\:\:\:\:\:\:\:\:\:\:\:\:j=0\:\:\:\:\:\:\:\\\:\:\\\:\left(1-\frac{{\upalpha\:}+1}{\mathcal{j}}\right){\text{C}}_{\mathcal{j}-1}^{{\upalpha\:}}\:\:\:\:\:\:\:\:\:\:\:\:j>0\:\:\:\:\end{array}\right.$$

#### Tuning of output layer weights

The weights in the output layer are updated using the fractional-order gradient descent method. Applying the general update rule, the output weights are optimized as:

15 $$\:{\mathcal{W}}_{\mathcal{m}}^{\mathcal{O}}\left({\upkappa\:}+1\right)=-{{\upeta\:}}_{{\mathcal{W}}_{\mathcal{m}}^{\mathcal{O}}}\frac{\partial\:\mathbb{E}\left({\upkappa\:}\right)}{\partial\:{\mathcal{W}}_{\mathcal{m}}^{\mathcal{O}}\left({\upkappa\:}\right)}{\mathcal{h}}^{{\upalpha\:}}-{\sum\:}_{\mathcal{j}=0}^{{\upkappa\:}}{\text{C}}_{\mathcal{j}}^{{\upalpha\:}}{\mathcal{W}}_{\mathcal{m}}^{\mathcal{O}}\left({\upkappa\:}\right)$$

where $\:{{\upeta\:}}_{{\mathcal{W}}_{\mathcal{m}}^{\mathcal{O}}}\:$is the learning rate for the output layer weights. The partial derivative of the error function is computed as:

16 $$\:\frac{\partial\:\mathbb{E}\left({\upkappa\:}\right)}{\partial\:{\mathcal{W}}_{\mathcal{m}}^{\mathcal{O}}\left({\upkappa\:}\right)}=\frac{\partial\:\mathbb{E}\left({\upkappa\:}\right)}{\partial\:{\mathcal{Y}}_{\text{F}\text{R}\text{N}\text{N}}\left({\upkappa\:}\right)}\cdot\:\frac{\partial\:{\mathcal{Y}}_{\text{F}\text{R}\text{N}\text{N}}\left({\upkappa\:}\right)}{\partial\:{\mathcal{W}}_{\mathcal{m}}^{\mathcal{O}}\left({\upkappa\:}\right)}=-\text{e}\left({\upkappa\:}\right)\cdot\:{\mathcal{Y}}_{\mathcal{m}}^{\mathcal{H}}\left({\upkappa\:}\right)$$

#### Tuning of hidden layer weights

The hidden layer weights $\:{\mathcal{W}}_{\mathcal{m}}^{\text{I}}$ and$\:{\mathcal{\:}\mathcal{W}}_{\mathcal{m}}^{\text{r}}$ are updated using the same fractional-order gradient descent approach:

17 $$\:{\mathcal{W}}_{\mathcal{m}}^{\text{I}}\left({\upkappa\:}+1\right)=-{{\upeta\:}}_{{\mathcal{W}}_{\mathcal{m}}^{\text{I}}}\frac{\partial\:\mathbb{E}\left({\upkappa\:}\right)}{\partial\:{\mathcal{W}}_{\mathcal{m}}^{\text{I}}\left({\upkappa\:}\right)}{\mathcal{h}}^{{\upalpha\:}}-{\sum\:}_{\mathcal{j}=0}^{{\upkappa\:}}{\text{C}}_{\mathcal{j}}^{{\upalpha\:}}{\mathcal{W}}_{\mathcal{m}}^{\text{I}}\left({\upkappa\:}\right)$$

18 $$\:{\mathcal{W}}_{\mathcal{m}}^{\text{r}}\left({\upkappa\:}+1\right)=-{{\upeta\:}}_{{\mathcal{W}}_{\mathcal{m}}^{\text{r}}}\frac{\partial\:\mathbb{E}\left({\upkappa\:}\right)}{\partial\:{\mathcal{W}}_{\mathcal{m}}^{\text{r}}\left({\upkappa\:}\right)}{\mathcal{h}}^{{\upalpha\:}}-{\sum\:}_{\mathcal{j}=0}^{{\upkappa\:}}{\text{C}}_{\mathcal{j}}^{{\upalpha\:}}{\mathcal{W}}_{\mathcal{m}}^{\text{r}}\left({\upkappa\:}\right)$$

where$\:\:\:{{\upeta\:}}_{{\mathcal{W}}_{\mathcal{m}}^{\text{I}}}$ and $\:{{\upeta\:}}_{{\mathcal{W}}_{\mathcal{m}}^{\text{r}}}\:$are the learning rates for the hidden layer weights. The partial derivatives of the error function are computed using the chain rule:

$$\:\frac{\partial\:\mathbb{E}\left({\upkappa\:}\right)}{\partial\:{\mathcal{W}}_{\mathcal{m}}^{\text{I}}\left({\upkappa\:}\right)}=\frac{\partial\:\mathbb{E}\left({\upkappa\:}\right)}{\partial\:{\mathcal{Y}}_{\text{F}\text{R}\text{N}\text{N}}\left({\upkappa\:}\right)}\cdot\:\frac{\partial\:{\mathcal{Y}}_{\text{F}\text{R}\text{N}\text{N}}\left({\upkappa\:}\right)}{\partial\:{\mathcal{Y}}_{\mathcal{m}}^{\mathcal{H}}\left({\upkappa\:}\right)}\cdot\:\frac{\partial\:{\mathcal{Y}}_{\mathcal{m}}^{\mathcal{H}}\left({\upkappa\:}\right)}{\partial\:{\text{n}\text{e}\text{t}}_{\mathcal{m}}^{\mathcal{H}}\left({\upkappa\:}\right)}\cdot\:\frac{\partial\:{\text{n}\text{e}\text{t}}_{\mathcal{m}}^{\mathcal{H}}\left({\upkappa\:}\right)}{\partial\:{\mathcal{W}}_{\mathcal{m}}^{\text{I}}\left({\upkappa\:}\right)}$$

19 $$\:=-\text{e}\left({\upkappa\:}\right)\cdot\:{\mathcal{W}}_{\mathcal{m}}^{\mathcal{O}}\cdot\:\stackrel{\prime }{\mathcal{F}}\left({\text{n}\text{e}\text{t}}_{\mathcal{m}}^{\mathcal{H}}\left({\upkappa\:}\right)\right)\cdot\:{\upchi\:}\left({\upkappa\:}\right)$$

$$\:\frac{\partial\:\mathbb{E}\left({\upkappa\:}\right)}{\:\partial\:{\mathcal{W}}_{\mathcal{m}}^{\text{r}}\left({\upkappa\:}\right)}=\frac{\partial\:\mathbb{E}\left({\upkappa\:}\right)}{\partial\:{\mathcal{Y}}_{\text{F}\text{R}\text{N}\text{N}}\left({\upkappa\:}\right)}\cdot\:\frac{\partial\:{\mathcal{Y}}_{\text{F}\text{R}\text{N}\text{N}}\left({\upkappa\:}\right)}{\partial\:{\mathcal{Y}}_{\mathcal{m}}^{\mathcal{H}}\left({\upkappa\:}\right)}\cdot\:\frac{\partial\:{\mathcal{Y}}_{\mathcal{m}}^{\mathcal{H}}\left({\upkappa\:}\right)}{\partial\:{\text{n}\text{e}\text{t}}_{\mathcal{m}}^{\mathcal{H}}\left({\upkappa\:}\right)}\cdot\:\frac{\partial\:{\text{n}\text{e}\text{t}}_{\mathcal{m}}^{\mathcal{H}}\left({\upkappa\:}\right)}{\partial\:{\mathcal{W}}_{\mathcal{m}}^{\text{r}}\left({\upkappa\:}\right)}$$

20 $$\:=-\text{e}\left({\upkappa\:}\right)\cdot\:{\mathcal{W}}_{\mathcal{m}}^{\mathcal{O}}\cdot\:\stackrel{\prime }{\mathcal{F}}\left({\text{n}\text{e}\text{t}}_{\mathcal{m}}^{\mathcal{H}}\left({\upkappa\:}\right)\right)\cdot\:{\mathcal{Y}}^{\mathcal{H}}({\upkappa\:}-1)$$

The integration of the Fractional-Order (FO) learning algorithm into the FRNN training process enables the model to account for historical information during weight updates. This memory-aware learning mechanism enhances both convergence speed and modeling accuracy, making it particularly effective for capturing the complex, nonlinear, and chaotic behaviors observed in real-world systems, such as diabetes progression.

The learning rate $\:{{\upeta\:}}_{{\uptheta\:}\:}$for FRNN parameters significantly affects system stability and convergence speed. A small value of $\:{{\upeta\:}}_{{\uptheta\:}\:}$ ensures convergence but increases training time. Conversely, selecting a large accelerates learning but may lead to system instability. Therefore, careful selection of the learning rate is required to balance learning speed and stability. In the following subsection, an investigation of the convergence analysis of the FO learning algorithm for the FRNN on the Lyapunov stability theorem is given^25^.

### Stability analysis of FRNN model

The following theorem establishes adaptive learning rates based on the Lyapunov stability method.

#### Theorem 1

The learning rates of the proposed FRNN model can be adapted to ensure system stability if the following condition holds:

21 $$\:0\le\:{{\upeta\:}}_{{\uptheta\:}}\le\:\frac{2}{{\left[\frac{\partial\:{\mathcal{Y}}_{\text{F}\text{R}\text{N}\text{N}}\left({\upkappa\:}\right)}{\partial\:{\uptheta\:}\left({\upkappa\:}\right)}\right]}^{2}}$$

#### Proof 1

Let the positive definite Lyapunov function be defined as:

22 $$\:\mathcal{L}\left({\upkappa\:}\right)=\frac{1}{2}{\text{e}}^{2}\left({\upkappa\:}\right)$$

Stability is guaranteed if:

23 $$\:\varDelta\:\mathcal{L}\left({\upkappa\:}\right)=\mathcal{L}\left({\upkappa\:}+1\right)-\mathcal{L}\left({\upkappa\:}\right)\le\:0$$

where $\:\varDelta\:\mathcal{L}\left({\upkappa\:}\right)$represents the change in the Lyapunov function, calculated as:

24 $$\:\varDelta\:\mathcal{L}\left({\upkappa\:}\right)=\frac{1}{2}\left({\text{e}}^{2}\left({\upkappa\:}+1\right)-{\text{e}}^{2}\left({\upkappa\:}\right)\right)$$

or equivalently:

25 $$\:\varDelta\:\mathcal{L}\left({\upkappa\:}\right)=\frac{1}{2}\left[\text{e}\left({\upkappa\:}+1\right)+\text{e}\left({\upkappa\:}\right)\right]\left[\text{e}\left({\upkappa\:}+1\right)-\text{e}\left({\upkappa\:}\right)\right]$$

Defining $\:\varDelta\:\text{e}\left({\upkappa\:}\right)=\text{e}\left({\upkappa\:}+1\right)-\text{e}\left({\upkappa\:}\right)$, Eq. (25) can be rewritten as.

26 $$\:\varDelta\:\mathcal{L}\left({\upkappa\:}\right)=\frac{1}{2}\left[\varDelta\:\text{e}\left({\upkappa\:}\right)+2\text{e}\left({\upkappa\:}\right)\right]\left[\varDelta\:\text{e}\left({\upkappa\:}\right)\right]=\varDelta\:\text{e}\left({\upkappa\:}\right)\left[\frac{1}{2}\varDelta\:\text{e}\left({\upkappa\:}\right)+\text{e}\left({\upkappa\:}\right)\right]$$

The optimal learning rate is inferred using the Taylor series expansion:

27 $$\:\text{e}\left({\upkappa\:}+1\right)=\text{e}\left({\upkappa\:}\right)+\frac{\partial\:\text{e}\left({\upkappa\:}\right)}{\partial\:{\uptheta\:}\left({\upkappa\:}\right)}+\text{{\rm\:H}}\text{{\rm\:O}}\text{{\rm\:T}}$$

Here $\:\partial\:{\uptheta\:}\left({\upkappa\:}\right)\:$refers to the parameters of FRNN identifier which should be tuned and HOT denotes higher order terms which can be neglected. Then Eq. (27) can be rewritten as.

28 $$\:\text{e}\left({\upkappa\:}+1\right)-\text{e}\left({\upkappa\:}\right)={\Delta\:}\text{e}\left({\upkappa\:}\right)=\frac{\partial\:\text{e}\left({\upkappa\:}\right)}{\partial\:{\uptheta\:}\left({\upkappa\:}\right)}{\Delta\:}{\uptheta\:}\left({\upkappa\:}\right)$$

Substituting Eq. (28) into Eq. (26):

29 $$\:\varDelta\:\mathcal{L}\left({\upkappa\:}\right)=\frac{\partial\:\text{e}\left({\upkappa\:}\right)}{\partial\:{\uptheta\:}\left({\upkappa\:}\right)}{\Delta\:}{\uptheta\:}\left({\upkappa\:}\right)\left[\frac{1}{2}\frac{\partial\:\text{e}\left({\upkappa\:}\right)}{\partial\:{\uptheta\:}\left({\upkappa\:}\right)}{\Delta\:}{\uptheta\:}\left({\upkappa\:}\right)+\text{e}\left({\upkappa\:}\right)\right]$$

For FRNN model, the parameter update rule is.

30 $$\:\varDelta\:{\uptheta\:}\left({\upkappa\:}\right)={{\upeta\:}}_{{\uptheta\:}}\text{e}\left({\upkappa\:}\right)\frac{\partial\:\text{e}\left({\upkappa\:}\right)}{\partial\:{\uptheta\:}\left({\upkappa\:}\right)}$$

Since the identification error is defined as: $\:\text{e}\left(\text{k}\right)=\mathcal{Y}\left({\upkappa\:}\right)-{\mathcal{Y}}_{\text{F}\text{R}\text{N}\text{N}}\left({\upkappa\:}\right)$, we obtain$\::\:\frac{\partial\:\text{e}\left({\upkappa\:}\right)}{\partial\:{\uptheta\:}\left({\upkappa\:}\right)}=-\frac{\partial\:{\mathcal{Y}}_{\text{F}\text{R}\text{N}\text{N}}\left({\upkappa\:}\right)}{\partial\:{\uptheta\:}\left({\upkappa\:}\right)}$. Thus, Eq. (30) can be rewritten as:

31 $$\:\varDelta\:{\uptheta\:}\left({\upkappa\:}\right)={-{\upeta\:}}_{{\uptheta\:}}\text{e}\left({\upkappa\:}\right)\frac{\partial\:{\mathcal{Y}}_{\text{F}\text{R}\text{N}\text{N}}\left({\upkappa\:}\right)}{\partial\:{\uptheta\:}\left({\upkappa\:}\right)}$$

Substituting Eq. (31) into Eq. (29), yields:

32 $$\:\varDelta\:\mathcal{L}\left({\upkappa\:}\right)={-{\upeta\:}}_{{\uptheta\:}}{\text{e}}^{2}\left({\upkappa\:}\right){\left(\frac{\partial\:{\mathcal{Y}}_{\text{F}\text{R}\text{N}\text{N}}\left({\upkappa\:}\right)}{\partial\:{\uptheta\:}\left({\upkappa\:}\right)}\right)}^{2}\left[1-\frac{{{\upeta\:}}_{{\uptheta\:}}}{2}{\left(\frac{\partial\:{\mathcal{Y}}_{\text{F}\text{R}\text{N}\text{N}}\left({\upkappa\:}\right)}{\partial\:{\uptheta\:}\left({\upkappa\:}\right)}\right)}^{2}\right]\:$$

For stability, $\:\varDelta\:\mathcal{L}\left({\upkappa\:}\right)\le\:0$, we have:

33 $$\:{-{\upeta\:}}_{{\uptheta\:}}{\text{e}}^{2}\left({\upkappa\:}\right){\left(\frac{\partial\:{\mathcal{Y}}_{\text{F}\text{R}\text{N}\text{N}}\left({\upkappa\:}\right)}{\partial\:{\uptheta\:}\left({\upkappa\:}\right)}\right)}^{2}\left[1-\frac{{{\upeta\:}}_{{\uptheta\:}}}{2}{\left(\frac{\partial\:{\mathcal{Y}}_{\text{F}\text{R}\text{N}\text{N}}\left({\upkappa\:}\right)}{\partial\:{\uptheta\:}\left({\upkappa\:}\right)}\right)}^{2}\right]\le\:0$$

Multiplying both sides by minus sign, we get:

34 $$\:{{\upeta\:}}_{{\uptheta\:}}{\text{e}}^{2}\left({\upkappa\:}\right){\left(\frac{\partial\:{\mathcal{Y}}_{\text{F}\text{R}\text{N}\text{N}}\left({\upkappa\:}\right)}{\partial\:{\uptheta\:}\left({\upkappa\:}\right)}\right)}^{2}\left[1-\frac{{{\upeta\:}}_{{\uptheta\:}}}{2}{\left(\frac{\partial\:{\mathcal{Y}}_{\text{F}\text{R}\text{N}\text{N}}\left({\upkappa\:}\right)}{\partial\:{\uptheta\:}\left({\upkappa\:}\right)}\right)}^{2}\right]\ge\:0$$

For the first term $\:{{\upeta\:}}_{{\uptheta\:}}{\text{e}}^{2}\left({\upkappa\:}\right){\left(\frac{\partial\:{\mathcal{Y}}_{\text{F}\text{R}\text{N}\text{N}}\left({\upkappa\:}\right)}{\partial\:{\uptheta\:}\left({\upkappa\:}\right)}\right)}^{2}$, we have:

35 $$\:{{\upeta\:}}_{{\uptheta\:}}\ge\:0$$

Also $\:\varDelta\:\mathcal{L}\left({\upkappa\:}\right)\le\:0$ if the second term in Eq. (34),$\:\:\left[1-\frac{{{\upeta\:}}_{{\uptheta\:}}}{2}{\left(\frac{\partial\:{\mathcal{Y}}_{\text{F}\text{R}\text{N}\text{N}}\left({\upkappa\:}\right)}{\partial\:{\uptheta\:}}\right)}^{2}\right]$, satisfies the condition.

36 $$\:\left[2-{{\upeta\:}}_{{\uptheta\:}}{\left(\frac{\partial\:{\mathcal{Y}}_{\text{F}\text{R}\text{N}\text{N}}\left({\upkappa\:}\right)}{\partial\:{\uptheta\:}\left({\upkappa\:}\right)}\right)}^{2}\right]\ge\:0$$

Thus we have.

37 $$\:{{\upeta\:}}_{{\uptheta\:}}\le\:\frac{2}{{\left(\frac{\partial\:{\mathcal{Y}}_{\text{F}\text{R}\text{N}\text{N}}\left({\upkappa\:}\right)}{\partial\:{\uptheta\:}\left({\upkappa\:}\right)}\right)}^{2}}\:$$

It follows from Eq. (35) and Eq. (36) that $\:\varDelta\:\mathcal{L}\left({\upkappa\:}\right)\:$is negative-definite if and only if the following asymptotic convergence condition is satisfied, ensuring $\:\varDelta\:\mathcal{L}\left({\upkappa\:}\right)\le\:0$.

38 $$\:0\le\:{{\upeta\:}}_{{\uptheta\:}}\le\:\frac{2}{{\left[\frac{\partial\:{\mathcal{Y}}_{\text{F}\text{R}\text{N}\text{N}}\left({\upkappa\:}\right)}{\partial\:{\uptheta\:}\left({\upkappa\:}\right)}\right]}^{2}}$$

Thus, the proof is complete. Consequently, the optimal adaptive learning rate for each tuned parameter of the developed FRNN model is given by:

39 $$\:{{\upeta\:}}_{{\uptheta\:}}=\frac{2}{{\left[\frac{\partial\:{\mathcal{Y}}_{\text{F}\text{R}\text{N}\text{N}}\left({\upkappa\:}\right)}{\partial\:{\uptheta\:}\left({\upkappa\:}\right)}\right]}^{2}}$$

## Simulation results

This section presents simulation results for the identification of chaotic diabetes system, demonstrating the efficiency of the FRNN model with the fractional-order (FO) learning algorithm. To assess the effectiveness of the FO learning algorithm and its impact on the performance of the FRNN model, the identification results of diabetes systems using FRNN-FO are compared with those obtained using alternative algorithms, including:

- FRNN based on gradient descent (FRNN-GD).
- Deep Feedforward Neural Network (DFNN)^29^.
- DRNN based on gradient descent (DRNN-GD).
- DRNN based on fractional-order learning (DRNN-FO).

For a fair comparison, all models are trained and tested using the same input/output dataset. The identification model’s weights are updated based on learning rates selected according to the adaptive rule derived in Eq. (39). To comprehensively evaluate performance, all models are compared using two metrics: Root Mean Square Error (RMSE) and FIT percent. The mathematical definitions of these metrics are given as:

40 $$\:\text{R}\text{M}\text{S}\text{E}=\sqrt{\frac{1}{\text{N}}\sum\:_{{\upkappa\:}}^{\text{N}}{(\mathcal{Y}\left({\upkappa\:}\right)-{\mathcal{Y}}_{\text{F}\text{R}\text{N}\text{N}}\left({\upkappa\:}\right))}^{2}}$$

41 $$FIT~\% = \left( {1 - \frac{{\left\| {{\mathcal{Y}} - {\mathcal{Y}}_{{FRNN}} } \right\|}}{{\left\| {{\mathcal{Y}} - mean\left( {\mathcal{Y}} \right)} \right\|}}} \right) \times 100$$

where $\:\mathcal{Y}\left({\upkappa\:}\right)$ is the actual system output, $\:{\mathcal{Y}}_{\text{F}\text{R}\text{N}\text{N}}\left({\upkappa\:}\right)$ is the predicted output of the FRNN model, and N is the total number of samples.

Based on the nonlinear model of the insulin–glucose regulatory system given in Eq. (1), the developed FRNN-FO model is designed to predict the dynamics of this chaotic system. The initial conditions are set as $\:{x}_{1}\left(0\right)=0.53$, $\:{x}_{2}\left(0\right)=1.31$, and $\:{x}_{3}\left(0\right)=1.03$. The network’s input layer consists of six nodes, and the input vector is defined as.

$$\:\left[{x}_{1}\right(k),{x}_{1}(k-1),{x}_{2}(k),{x}_{2}(k-1),{x}_{3}(k),{x}_{3}(k-1\left)\right].$$

The output layer contains a single node corresponding to the glucose concentration, $\:{x}_{2}(k+1)$. The hidden layer comprises five neurons, and all network weights in the hidden and output layers are initialized randomly. The model hyperparameters and training settings are summarized in Table 2.

**Table 2:** Model hyperparameters and training settings.

Network	NO. of layers	Neurons per layer	Initialization Method	Activation Function	Fractional Order Derivative($\:\alpha\:$)	Learning rate	Implementation Platform
DRNN-GD	3	6-5-1	Random initialization	tanh	-	$$\:\left\{\begin{array}{c}{\eta\:}_{i}=0.1\:\\\:{\eta\:}_{h}=0.21\\\:{\eta\:}_{o}=0.1\end{array}\right.$$	Matlab 2014a
DRNN-FO	3	6-5-1	Random initialization	tanh	0.3999	$$\:\left\{\begin{array}{c}{\eta\:}_{i}=0.77\\\:{\eta\:}_{h}=0.55\\\:{\eta\:}_{o}=0.97\end{array}\right.$$	Matlab 2014a
DFNN	3	6–28–1	Random initialization	sigmoid	-	$$\:\left\{\begin{array}{c}{\eta\:}_{i}=0.01\\\:{\eta\:}_{h}=0.01\\\:{\eta\:}_{o}=0.01\end{array}\right.$$	Matlab 2014a
FRNN-GD	3	6–5–1	Random initialization	tanh	-	$$\:\left\{\begin{array}{c}{\eta\:}_{i}=0.1\\\:{\eta\:}_{h}=0.05\\\:{\eta\:}_{o}=0.95\end{array}\right.$$	Matlab 2014a
FRNN-FO	3	6-5-1	Random initialization	tanh	0.3109	$$\:\left\{\begin{array}{c}{\eta\:}_{i}=0.05\\\:{\eta\:}_{h}=0.05\\\:{\eta\:}_{o}=0.9\end{array}\right.$$	Matlab 2014a

In this section, the developed FRNN-FO algorithm is applied to simulate the chaotic dynamics of the disorders which are mentioned in Sect. 2, and its responses are compared with those of the FRNN-GD, DFNN, DRNN-GD, and DRNN-FO algorithms. An input-output dataset of 4,000 samples is generated using different values of the insulin-glucose regulatory system parameters. During the modeling phase, a dataset of 2,800 samples (70%) is used for training, while the remaining 1,200 samples (30%) are reserved for testing.

### Type 2 diabetes case

**Fig. 3:**
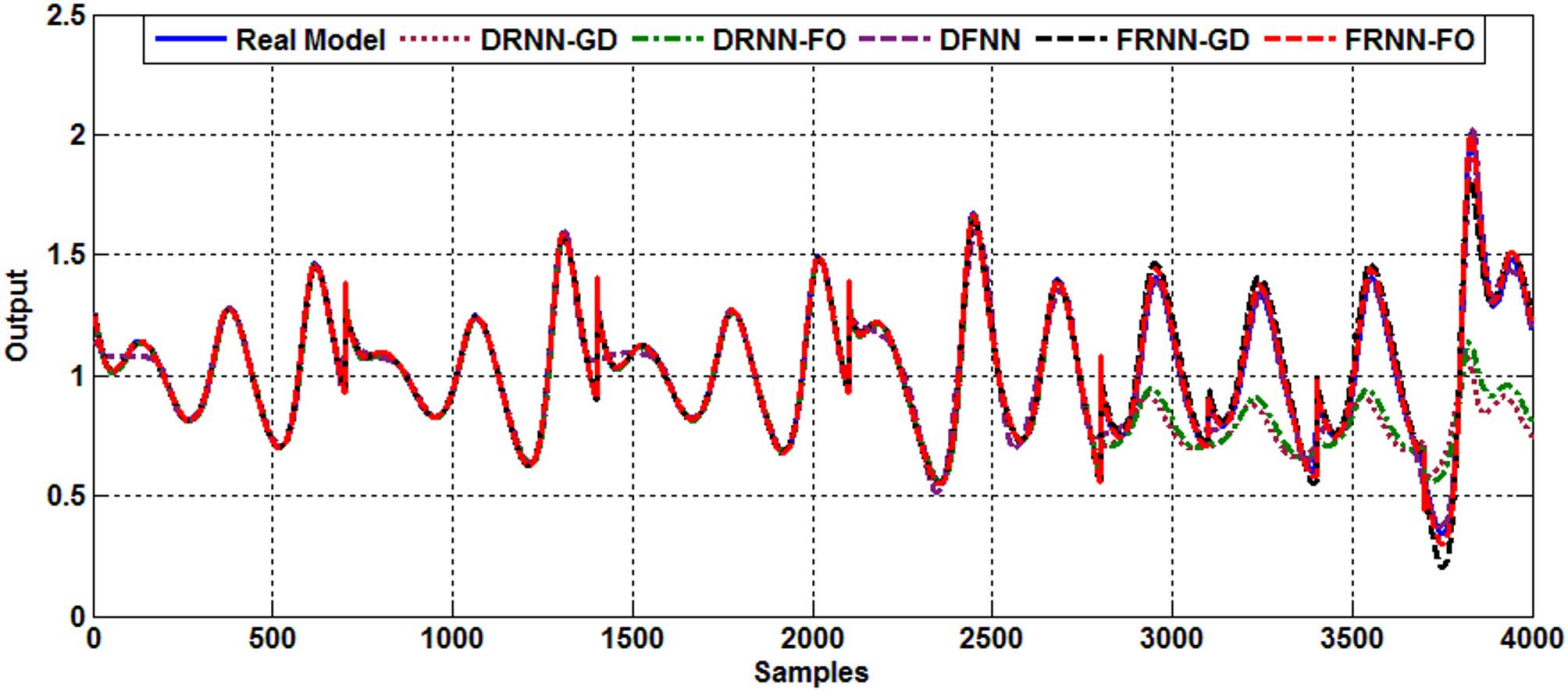
Output response of Type 2 diabetes.

The insulin-glucose regulatory system is stimulated using four different values of the $\:{\text{w}}_{8}$ parameter ($\:{\text{w}}_{8}=0.5,\:0.2,\:0.42\:\text{a}\text{n}\text{d}\:0.3$) to represent four cases of type 2 diabetes. For each w8 ​ value, an input-output dataset of 1,000 samples is generated during the modeling phase to identify type 2 diabetes in the insulin-glucose regulatory system using FRNN. Figure 3 illustrates the predicted responses of FRNN-FO and other models for the chaotic behavior of type 2 diabetes under different parameter settings. The first 2,800 samples represent the behavior of the comparative models during the training phase, while the remaining 1,400 samples correspond to the test phase responses. The chaotic behavior of type 2 diabetes is successfully identified using the FRNN-FO algorithm, which demonstrates superior performance compared to the other models. Table 3 provides a comparative evaluation of all considered models by presenting the measured performance indices. As shown in Table 3, the proposed FRNN-FO model achieves better results in both the training and test phases, exhibiting lower RMSE values and higher FIT% values than the other models.

**Table 3:** Comparative performance of models for type 2 Diabetes.

Model	DRNN-GD	DRNN-FO	DFNN	FRNN-GD	FRNN-FO
RMSE (Training)	0.0330	0.0333	0.0352	0.0076	0.0059
Fit % (Training)	96.5183	97.5629	93.331	98.1545	98.7968
RMSE (Test)	0.1920	0.1712	0.0414	0.0363	0.0128
Fit % (Test)	61.7300	65.3968	95.8507	92.2547	97.3861

### Hypoglycemia case

**Fig. 4:**
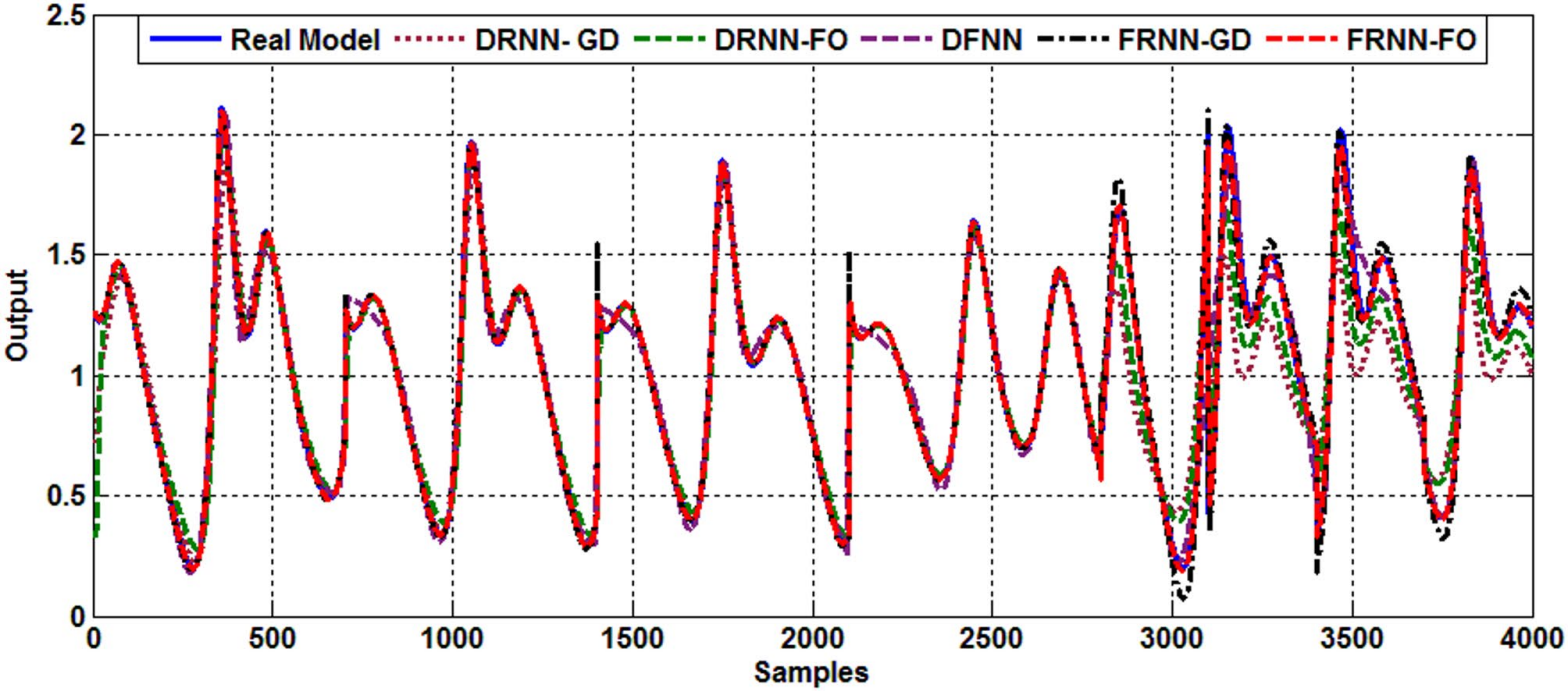
Output response of hypoglycemia.

In this case, the developed model is designed to predict hypoglycemia condition with chaotic behavior, which occurs when the parameter $\:{\text{w}}_{1}$, representing the rate of insulin decay, is decreased. Like the previous case, a dataset of 4,000 samples is generated under four different cases of hypoglycemia, corresponding to different values of $\:{\text{w}}_{1}$ ($\:{\text{w}}_{1}=1.3,\:1.7,\:1.8\:\text{a}\text{n}\text{d}\:2.04$). Figure 4 presents the identified system for this disorder across all models. As evident from the system responses, the proposed FRNN-FO algorithm accurately identifies the chaotic behavior of hypoglycemia in the insulin-glucose regulatory system, outperforming other models, particularly in the testing phase. Additionally, the superiority of the FRNN-FO algorithm is confirmed by the RMSE and FIT% results in Table 4, which demonstrate its superior performance.

**Table 4:** Comparative performance of models for hypoglycemia.

Model	DRNN-GD	DRNN-FO	DFNN	FRNN-GD	FRNN-FO
RMSE (Training)	0.0706	0.0743	0.0479	0.0102	0.0119
Fit % (Training)	97.9995	98.9888	91.0435	98.9995	99.4354
RMSE (Test)	0.1603	0.1059	0.1436	0.0431	0.0221
Fit % (Test)	79.0224	89.5723	90.9138	91.5794	97.6159

### Hyperinsulinemia case

**Fig. 5:**
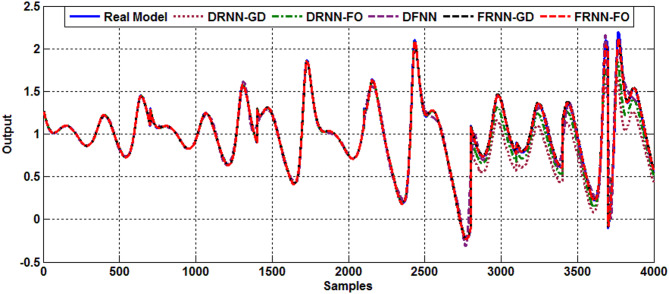
Output response of hyperinsulinemia.

In the insulin-glucose regulatory system model defined in Eq. (1), the parameter $\:{\text{w}}_{7}$ represents the amplified rate of insulin secretion by β-cells. The system remains stable for small values of $\:{\text{w}}_{7}$​; however, as this parameter increases, the system transitions into chaotic behavior. Similar previous cases, 4000 samples are generated for different high values of $\:{\text{w}}_{7}$ ($\:{\text{w}}_{7}=1.9,\:2.01,\:2.5\:\text{a}\text{n}\text{d}\:3.1$) to simulate the chaotic behavior associated with hyperinsulinemia disorder. The modeling results for these cases during both training and testing phases are illustrated in Fig. 5. As evident from the response, the proposed FRNN-FO algorithm demonstrates superior performance in effectively modeling the chaotic behavior of hyperinsulinemia within the insulin-glucose regulatory system compared to other identification techniques. Table 5 presents the RMSE and FIT% values, confirming that the proposed identification technique achieves the lowest RMSE and highest FIT% values in both training and testing phases.

**Table 5:** Comparative performance of models for hyperinsulinemia.

Model	DRNN-GD	DRNN-FO	DFNN	FRNN-GD	FRNN-FO
RMSE (Training)	0.0181	0.0167	0.0454	0.0082	0.0056
Fit % (Training)	98.8791	99.0204	94.2309	98.5768	99.0597
RMSE (Test)	0.1373	0.0733	0.1150	0.0204	0.0157
Fit % (Test)	69.4335	81.5067	91.6003	89.8901	97.4341

### Type 1 diabetes case

**Fig. 6:**
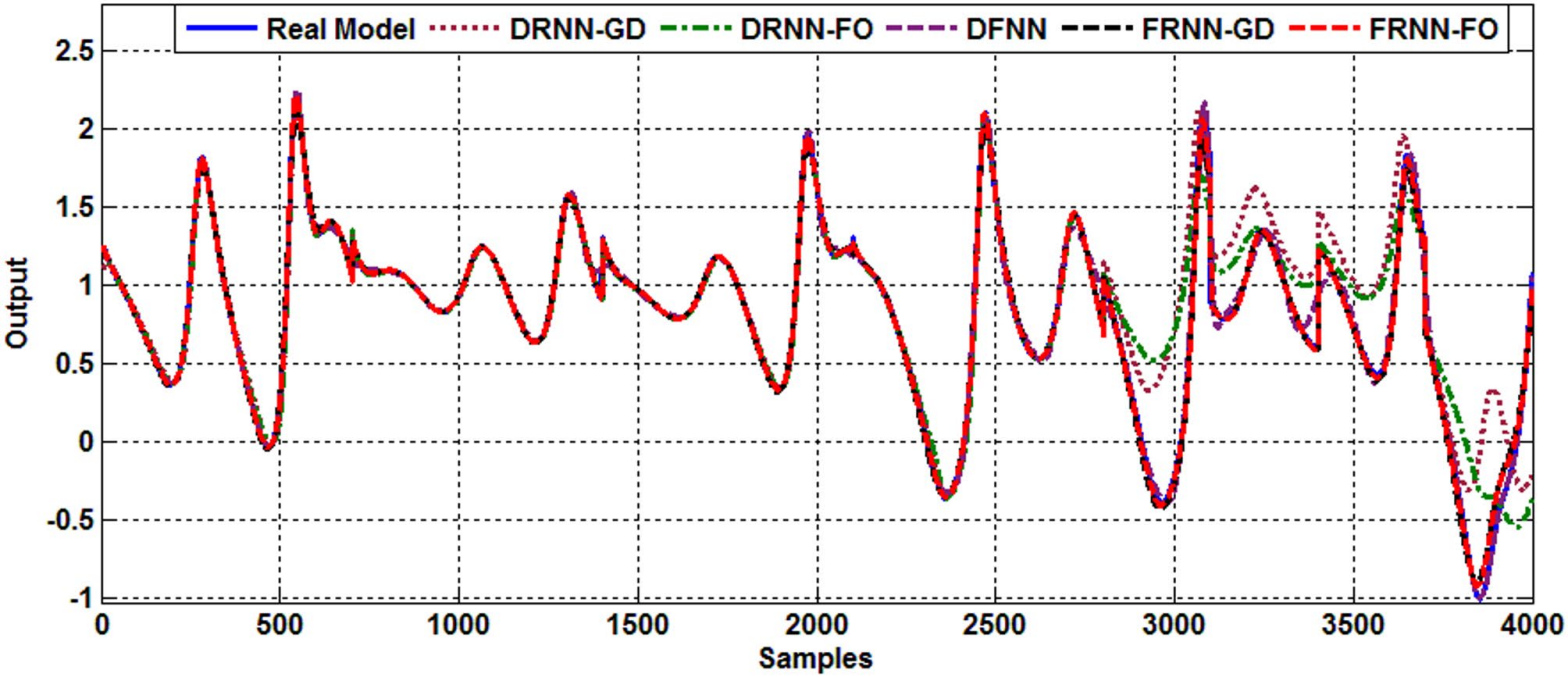
Output response of type 1 diabetes.

In this simulation, when the rate of increase in the population density of β-cells, represented by $\:{\text{w}}_{15}$ in Eq. (1), decreases, the pancreas is unable to secrete sufficient insulin to regulate glucose levels, leading to a disruption in the insulin-glucose regulatory system—characteristic of type 1 diabetes. Under these conditions, the system exhibits chaotic behavior at lower values of $\:{\text{w}}_{15}$​. Training and testing data are generated based on different values of $\:{\text{w}}_{15}$ ($\:{\text{w}}_{15}=0.16,\:0.3,\:0.25\:\text{a}\text{n}\text{d}\:0.13$) following the same approach as in previous simulation cases. The results of the proposed scheme, along with those of the comparative models for the identified type 1 diabetes case, are presented in Fig. 6. The results clearly show that the FRNN-FO algorithm effectively captures the chaotic behavior associated with type 1 diabetes in the insulin-glucose regulatory system, surpassing other identification methods. Table 6 presents the RMSE and FIT% values for all models, highlighting the proposed model’s superior performance with lower RMSE and higher FIT% values.

**Table 6:** Comparative performance of models for type 1 diabetes.

Model	DRNN-GD	DRNN-FO	DFNN	FRNN-GD	FRNN-FO
RMSE (Training)	0.0384	0.0378	0.0345	0.0172	0.0073
Fit % (Training)	95.8432	96.5111	95.5040	97.1683	99.2593
RMSE (Test)	0.2699	0.2475	0.0743	0.0395	0.0307
Fit % (Test)	50.6540	71.2072	93.0047	93.1618	95.4778

### Uncertainty analysis

 Regarding the proposed FRNN-FO model focuses on point prediction of the chaotic behavior of insulin-glucose regulatory system under various diabetes-related disorders, evaluation of the uncertainty associated with these predictions is viewed as an important topic. From this point of view, Quantile Regression (QR) is performed to estimate the uncertainty of the proposed FRNN-FO model by evaluating the model residual (i.e. modeling error) that can reflect the different sources of the uncertainty^30–32^. The processing of this method can be described as follows.

Quantile regression enables the construction of prediction intervals by modeling the conditional quantiles of the target variable’s distribution. QR method is processed with assuming a linear relationship between the model predicted outputs and the observed output of the insulin-glucose regulatory system. For each specified quantile level $\:\tau\:$, the fitted model is described by the following linear relation as:

42 $$\:\mathcal{y}={\mathcal{a}}_{\tau\:}\stackrel{\sim}{\mathcal{y}}+{\mathcal{b}}_{\tau\:}$$

where $\:{\mathcal{a}}_{\tau\:},{\mathcal{b}}_{\tau\:}$ are the slop and intercept of the QR, respectively. These parameters are calculated by minimizing the following sum of residuals:

43 $$\:\text{m}\text{i}\text{n}\sum\:_{\text{k}=1}^{\text{K}}{{\uprho\:}}_{\text{k}}{\left({\mathcal{y}}_{k}-({\mathcal{a}}_{\tau\:}{\stackrel{\sim}{\mathcal{y}}}_{k}+{\mathcal{b}}_{\tau\:})\right)}^{2}$$

where $\:{\mathcal{y}}_{k}$ and $\:{\stackrel{\sim}{\mathcal{y}}}_{k}$ are the observed and the predicted outputs of the proposed FRNN-FO model at sampling instant *k*, respectively. $\:{{\uprho\:}}_{\text{k}}$ is the QR function of the $\:{{\uptau\:}}^{\text{t}\text{h}}$ quantile given as:

44 $$\:{{\uprho\:}}_{\text{k}}\left({{\upepsilon\:}}_{\text{k}}\right)=\left\{\begin{array}{c}(\tau\:-1){{\upepsilon\:}}_{\text{k}}\:\:\:\:\:\:\:\:{{\upepsilon\:}}_{\text{k}}<0\:\\\:\left({\uptau\:}\right){{\upepsilon\:}}_{\text{k}}\:\:\:\:\:\:\:\:\:\:\:\:\:\:\:\:{{\upepsilon\:}}_{\text{k}}\ge\:0\:\end{array}\right.\:$$

The uncertainty of the proposed model is estimated and the quality of the constructed prediction intervals is evaluated using two statistical metrics; Prediction Interval Coverage Probability (PICP) and Mean Prediction Interval (MPI).

Firstly, the Prediction Interval Coverage Probability (PICP) can be calculated as:

45 $$\:\text{P}\text{I}\text{C}\text{P}=\sum\:_{\text{k}=1}^{\text{K}}{{\upzeta\:}}_{\text{k}}\:,\:\:\:\:\:\:\:\:\:\:\:\:{{\upzeta\:}}_{\text{k}}=\left\{\begin{array}{c}1,\:{\text{P}}_{\text{k}}^{\text{l}\text{o}\text{w}\text{e}\text{r}}<{\mathcal{y}}_{k}<{\text{P}}_{\text{k}}^{\text{u}\text{p}\text{p}\text{e}\text{r}}\:\\\:0,\:otherwise\end{array}\right.$$

$\:{\text{P}}_{\text{k}}^{\text{l}\text{o}\text{w}\text{e}\text{r}}$ and $\:{\text{P}}_{\text{k}}^{\text{u}\text{p}\text{p}\text{e}\text{r}}$ are the lower and upper limit of the predictive intervals, respectively. Actually, PICP measures the probability of the observed values which fall within the prediction intervals (i.e. between 5% and 95%) or 90% confidence level. The good predicted model should be with a PICP near the confidence limit (90% with some tolerance).

Secondly, the mean prediction interval (MPI) defines the average width of the prediction intervals and it is calculated as.

46 $$\:\text{M}\text{P}\text{I}=\sum\:_{\text{k}=1}^{\text{K}}\left({\text{P}}_{\text{k}}^{\text{u}\text{p}\text{p}\text{e}\text{r}}-{\text{P}}_{\text{k}}^{\text{l}\text{o}\text{w}\text{e}\text{r}}\right)\:\:$$

The small value of MPI reflects sharper (narrower) prediction intervals and the large MPI values indicate greater predictive uncertainty.

For uncertainty estimation and analysis, the above described QR approach has been applied for the proposed FRNN-FO model. The QR based uncertainty analysis was conducted during the training and test phases and it is evaluated for the different four disorders of the insulin-glucose regulatory system as described in Sect. 4.

**Fig. 7:**
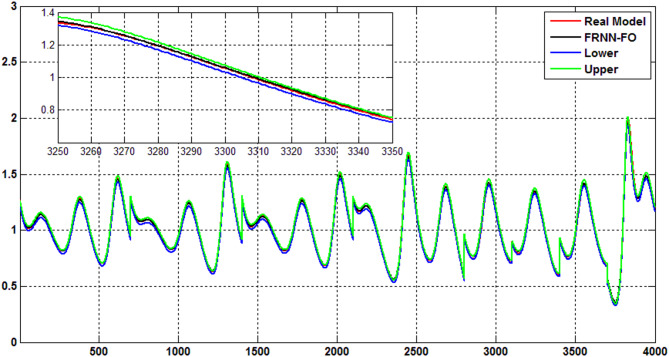
The uncertainty bands predicted using QR method for the proposed FRNN-FO model of Type 2 diabetes.

**Fig. 8:**
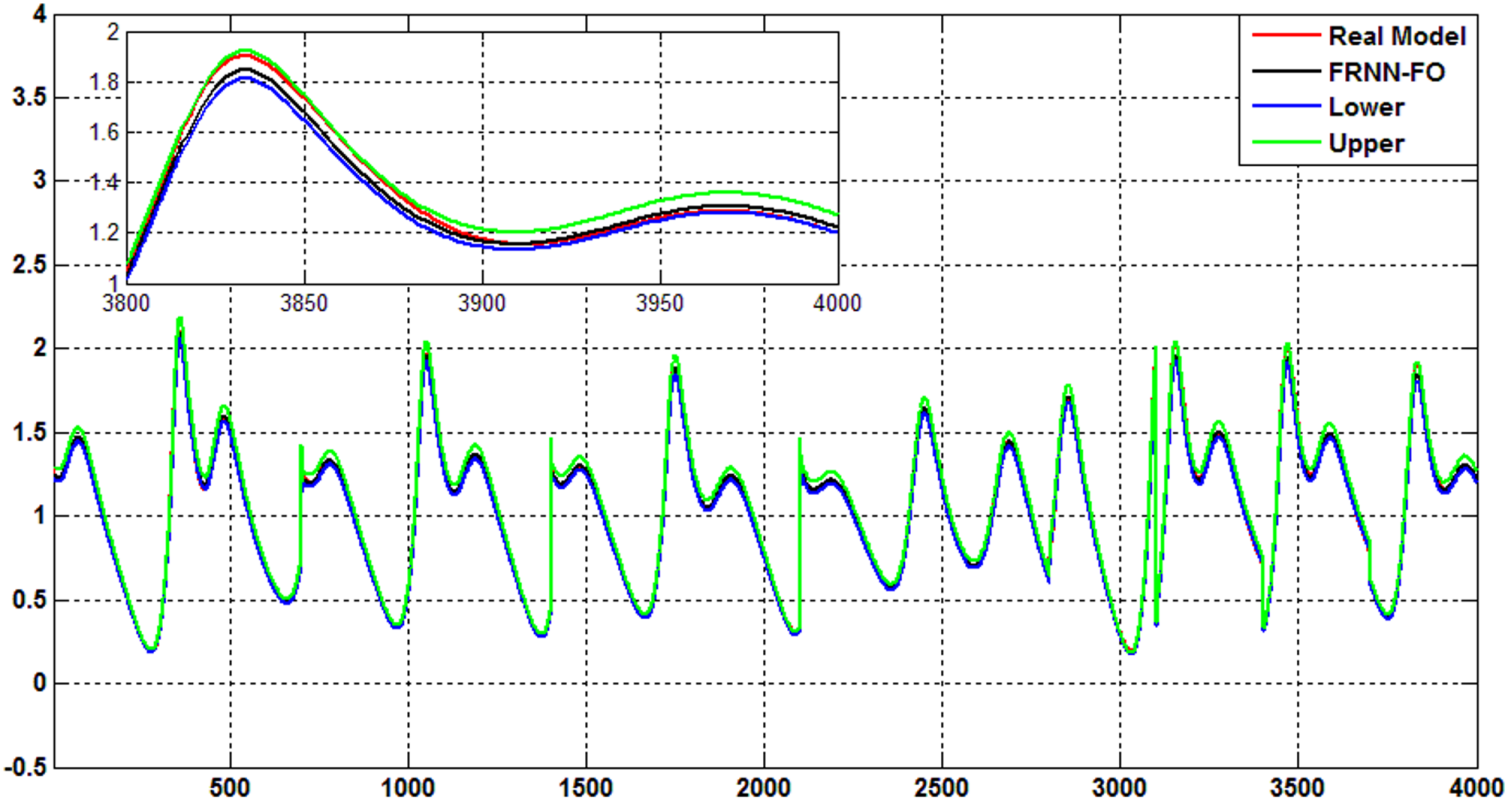
The uncertainty bands predicted using QR method for the proposed FRNN-FO model of hypoglycemia.

**Fig. 9:**
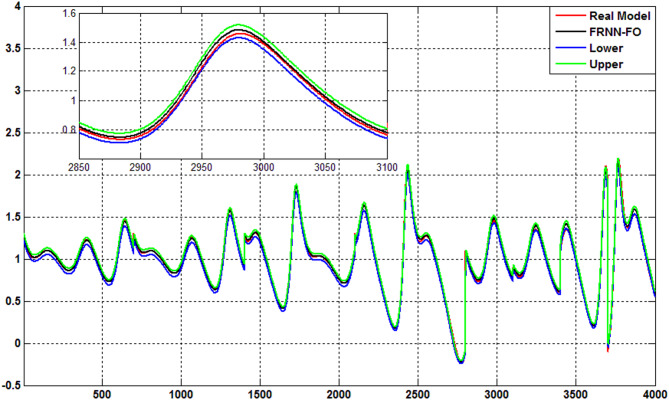
The uncertainty bands predicted using QR method for the proposed FRNN-FO model of hyperinsulinemia.

**Fig. 10:**
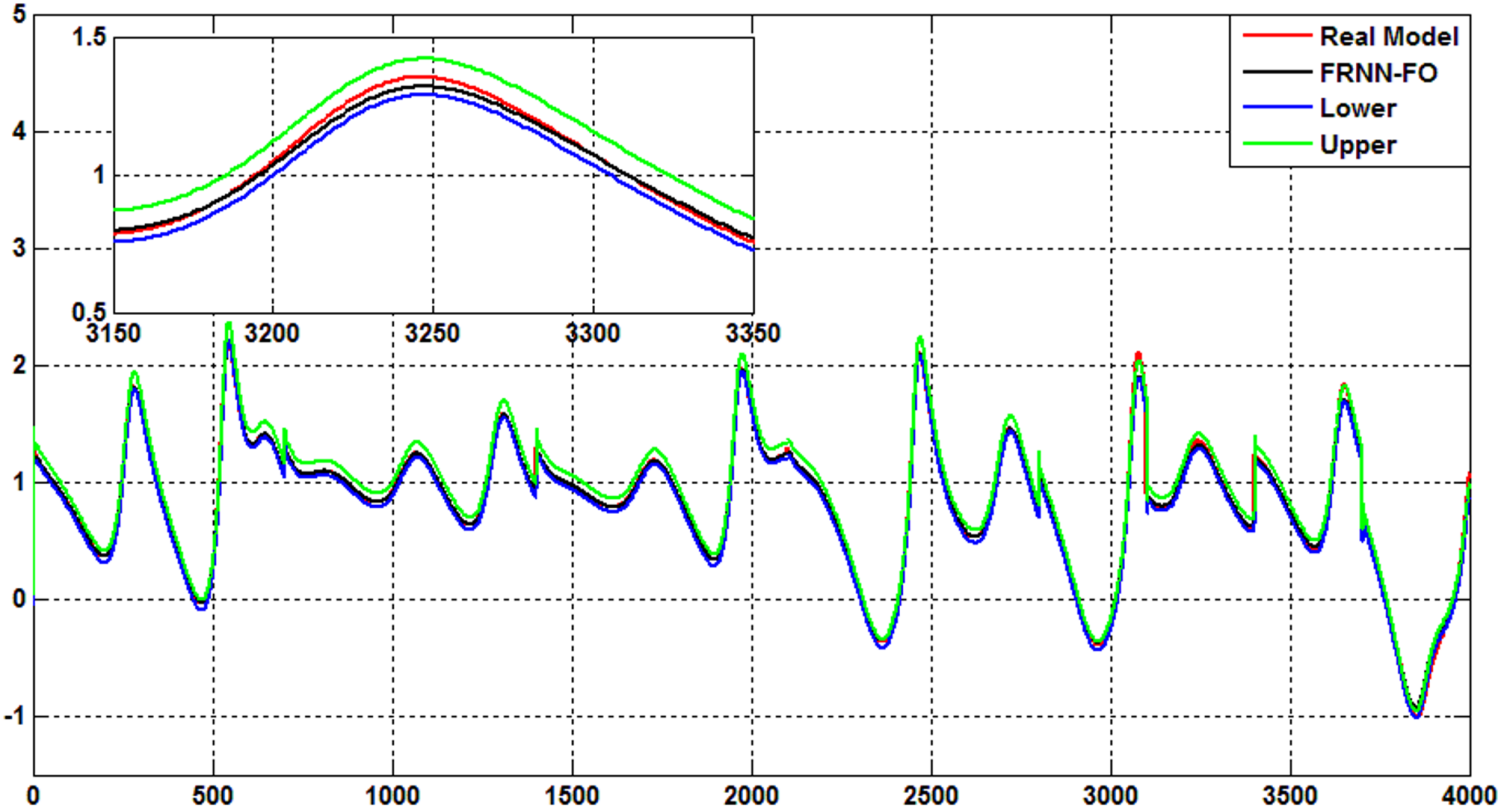
The uncertainty bands predicted using QR method for the proposed FRNN-FO model of Type 1 diabetes.

The uncertainty bands predicted of the proposed FRNN-FO model using the Quantile Regression (QR) method for type 2 diabetes, hyperinsulinemia, hypoglycemia, and type 1 diabetes are indicated in Figs. 7, 8, 9 and 10. As evident the uncertainty estimation using the QR method demonstrates the superior performance of the proposed FRNN-FO with low uncertainty values in the prediction of all chaotic cases within the insulin-glucose regulatory systemduring the training and test phases.

Table 7 presents the values of PICP and MPI with all considered models in a comparative manner for all different diabetes-related conditions. It is observed that, all modeling structures exhibit PICP values that closely to 90% confidence for both training and test phases. However, the measured MPI values are consistently lower with the proposed FRNN-FO approach for type 2 diabetes, hyperinsulinemia, hypoglycemia, and type 1 diabetes. This indicates the effectiveness of the proposed model to produce high precise predictions with narrow width of the prediction intervals.

**Table 7:** The PICP and the MPI values for the four models.

Criterion		Training		Test	
Diabetes case	Model	PICP	MPI	PICP	MPI
Type 2	DRNN-GD	0.8996	0.0597	0.9000	0.5725
	DRNN-FO	0.8999	0.0413	0.8996	0.2894
	DFNN	0.9000	0.1195	0.9000	0.1406
	FRNN-GD	0.9004	0.0273	0.9001	0.1999
	FRNN-FO	0.9010	0.0252	0.9003	0.0583
Hypoglycemia	DRNN-GD	0.9000	0.0533	0.8992	1.0459
	DRNN-FO	0.9001	0.0467	0.8996	0.2785
	DFNN	0.9002	0.1377	0.9000	0.3319
	FRNN-GD	0.9003	0.0376	0.9000	0.1963
	FRNN-FO	0.9007	0.0289	0.9008	0.0771
Hyperinsulinemia	DRNN-GD	0.8996	0.0448	0.8997	0.2929
	DRNN-FO	0.9000	0.0423	0.8999	0.2216
	DFNN	0.8999	0.1009	0.9000	0.2438
	FRNN-GD	0.8997	0.0293	0.9001	0.1119
	FRNN-FO	0.9004	0.0209	0.9008	0.1024
Type 1	DRNN-GD	0.9000	0.0711	0.8992	0.8086
	DRNN-FO	0.9000	0.0603	0.8997	0.5788
	DFNN	0.8993	0.0924	0.9001	0.2033
	FRNN-GD	0.9011	0.0563	0.9003	0.1816
	FRNN-FO	0.9020	0.0403	0.9009	0.1367

### Statistical analysis

**Fig. 11:**
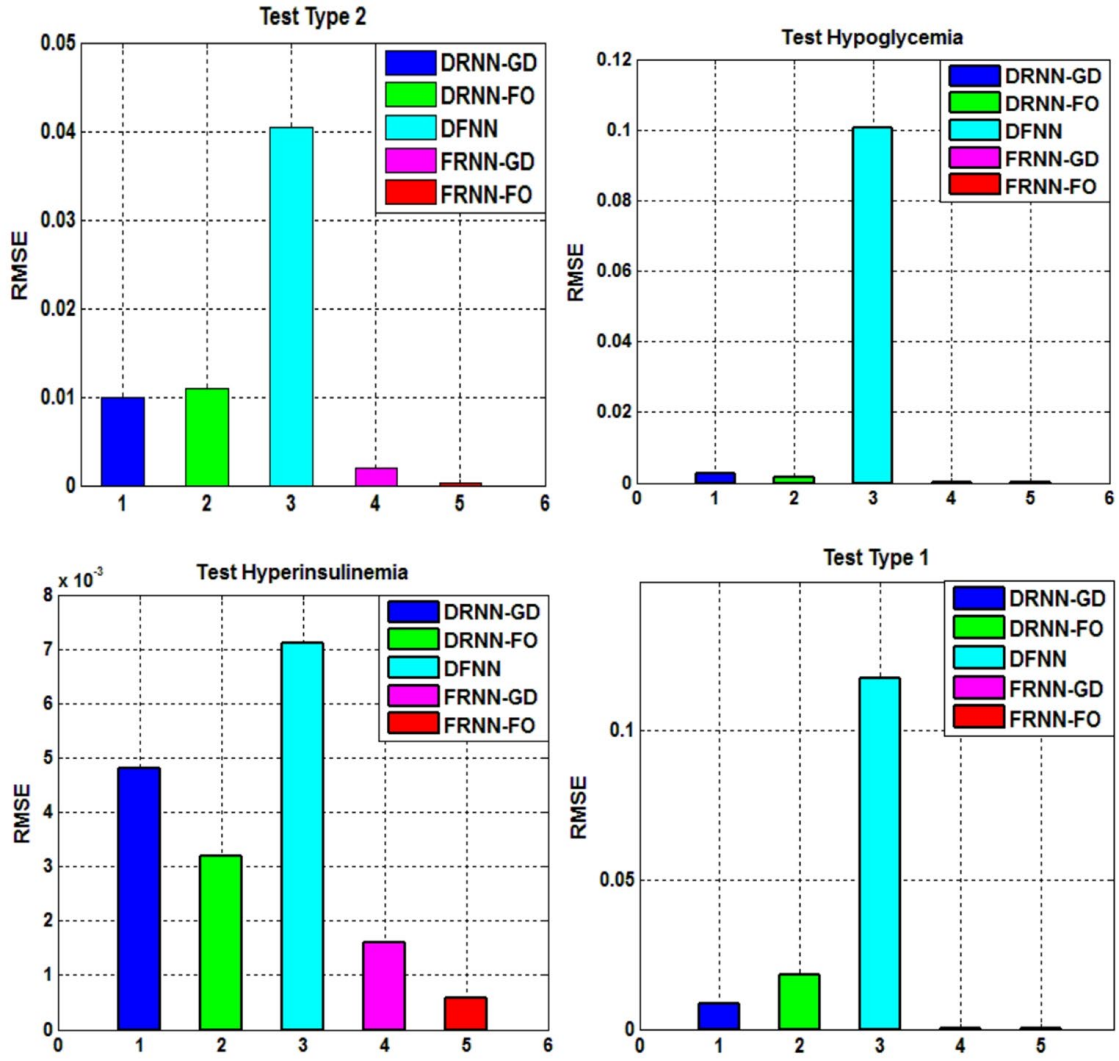
Histogram representation of the statistical analysis results (RMSE) for diabetes cases.

**Fig. 12:**
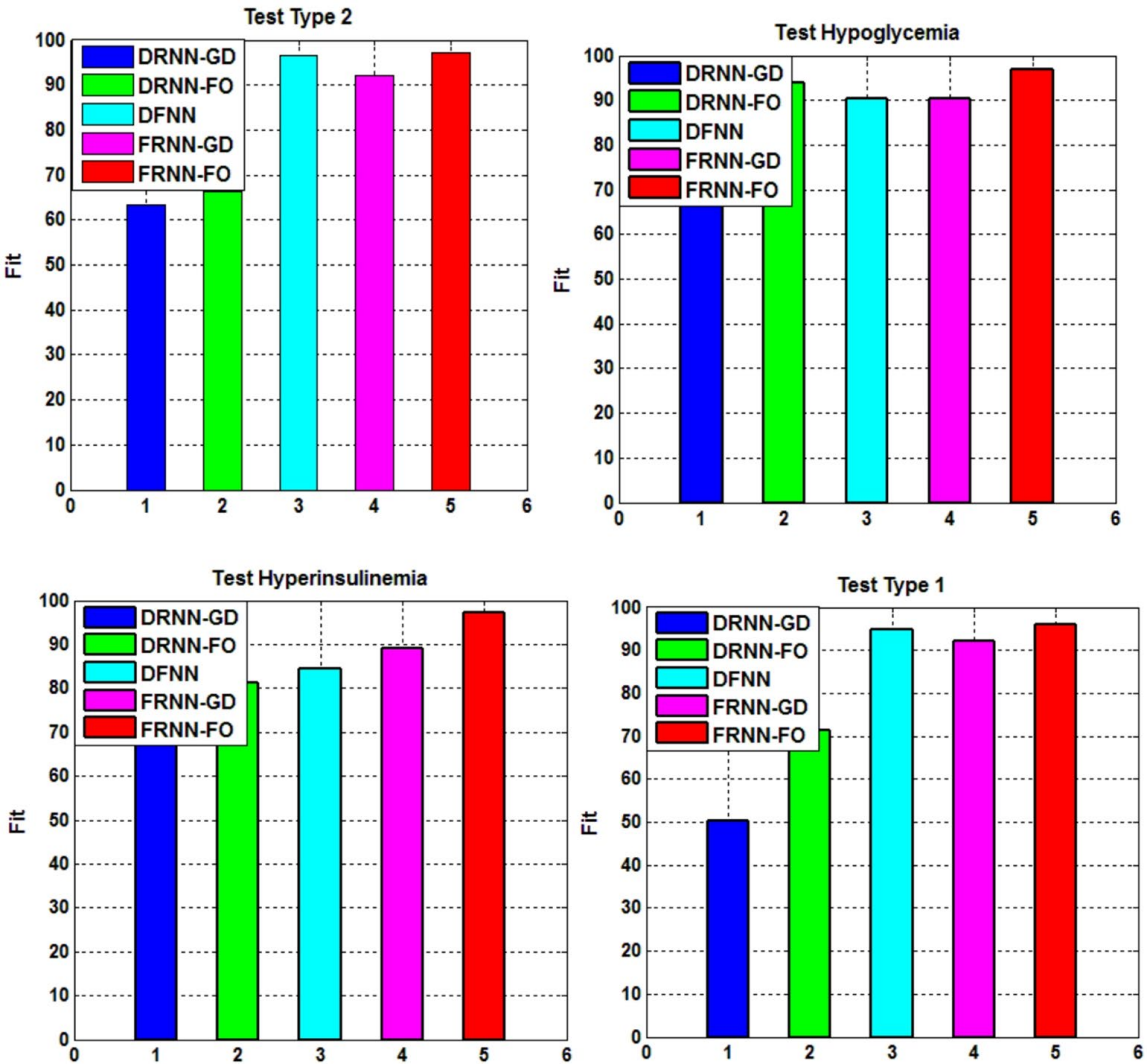
Histogram representation of the statistical analysis results (Fit%) for diabetes cases.

To ensure the robustness and reliability of the proposed FRNN-FO model, a comprehensive statistical analysis is conducted on error performance measurements, specifically RMSE and FIT%. This analysis is performed over ten independent runs for each simulation case to validate the results. Tables 8 and 9 present the statistical analysis results for each case based on three key metrics: best value, worst value and standard deviation of both RMSE and FIT%. Each table compares the performance of FRNN-FO against benchmark models, including DRNN-GD, DRNN-FO, DFNN, and FRNN-GD. Additionally, Figs. 11 and 12 provide 2D histogram representations, visually depicting the error distribution across multiple trials.

The statistical values in Tables 8 and 9 highlight the superior performance of FRNN-FO across different diabetes-related conditions. Key observations include:

- Lowest RMSE in training and testing: FRNN-FO demonstrates superior approximation capability and generalization. For instance, in Type 1 Diabetes (Table 7), FRNN-FO attains an RMSE of 0.00024 in testing, significantly outperforming DRNN-GD (0.0087), DFNN (0.1176) and DRNN-FO (0.0180).
- Highest FIT% values: The proposed model accurately captures the underlying system dynamics. In Type 2 Diabetes (Table 8), FRNN-FO achieves a FIT% of 97.13%, surpassing FRNN-GD (92.25%) and DRNN-FO (66.19%).
- Lower standard deviation values: FRNN-FO maintains the lowest error variance, ensuring greater reliability across multiple runs. Competing models exhibit higher variability and inconsistent results, as indicated in Tables 8 and 9.

The histograms in Figs. 11 and 12 confirm that FRNN-FO’s error values are more concentrated around the lower RMSE range, indicating greater stability. In contrast, competing models such as DRNN-GD and DRNN-FO exhibit wider error distributions, suggesting potential performance fluctuations. These graphical insights complement the tabulated results, reinforcing FRNN-FO’s robustness in identifying chaotic diabetes dynamics with superior accuracy.

**Table 8:** Statistical analysis results of RMSE for diabetes Cases.

Criterion		RMSE_ Training			RMSE_ Test		
Diabetes case	Model	Best value	Worst value	Standard deviation	Best value	Worst value	Standard deviation
Type 2	DRNN-GD	0.00011	0.0003	0.7545 × 10⁻⁴	0.0099	0.0099	0.0008 × 10⁻⁴
	DRNN-FO	0.0002	0.0002	0.1327 × 10⁻³	0.0110	0.0110	0.0005 × 10⁻⁴
	DFNN	0.0333	0.0428	0.0020	0.0404	0.0423	0.0015
	FRNN-GD	0.0001	0.0011	0.3086 × 10⁻³	0.0019	0.0019	0.00003 × 10⁻³
	FRNN-FO	0.0283 × 10⁻³	0.8088 × 10⁻³	0.2764 × 10⁻³	0.2565 × 10⁻³	0.2565 × 10⁻³	0.00001 × 10⁻³
Hypoglycemia	DRNN-GD	0.0001	0.0003	0.7774 × 10⁻⁴	0.0027	0.0027	0.0009 × 10⁻⁴
	DRNN-FO	0.0001	0.0007	0.2564 × 10⁻³	0.0016	0.0016	0.0007 × 10⁻³
	DFNN	0.0444	0.0795	0.0050	0.1437	0.1006	0.0071
	FRNN-GD	0.0025 × 10⁻³	0.4388 × 10⁻³	0.1844 × 10⁻³	0.1820 × 10⁻³	0.1820 × 10⁻³	0.0005 × 10⁻⁴
	FRNN-FO	0.0001 × 10⁻³	0.0033 × 10⁻³	0.7853 × 10⁻⁴	0.5332 × 10⁻⁴	0.5332 × 10⁻⁴	0.0002 × 10⁻⁴
Hyperinsulinemia	DRNN-GD	0.0000	0.0004	0.7926 × 10⁻⁴	0.0048	0.0048	0.0041 × 10⁻⁴
	DRNN-FO	0.0000	0.0005	0.1238 × 10⁻³	0.0032	0.0032	0.0032 × 10⁻³
	DFNN	0.0292	0.1475	0.0314	0.1058	0.1565	0.0364
	FRNN-GD	0.0002	0.0004	0.8038 × 10⁻⁴	0.0016	0.0016	0.0001 × 10⁻³
	FRNN-FO	0.5698 × 10⁻³	0.8710 × 10⁻³	0.9567 × 10⁻⁴	0.5838 × 10⁻³	0.5838 × 10⁻³	0.0002 × 10⁻⁴
Type 1	DRNN-GD	0.0001	0.0002	0.6501 × 10⁻⁴	0.0087	0.0087	0.0095 × 10⁻⁴
	DRNN-FO	0.00001	0.0001	0.3297 × 10⁻⁴	0.0180	0.0180	0.0061 × 10⁻⁴
	DFNN	0.0351	1.0856	0.0204	0.0806	0.1176	0.0231
	FRNN-GD	0.0368 × 10⁻³	0.7570 × 10⁻³	0.2312 × 10⁻³	0.4499 × 10⁻³	0.4499 × 10⁻³	0.0004 × 10⁻³
	FRNN-FO	0.000001	0.0005	0.1579 × 10⁻³	0.00024	0.00024	0.0001 × 10⁻³

**Table 9:** Statistical analysis results of Fit% for diabetes cases.

Criterion		Fit %_ Training			Fit %_ Test		
Diabetes case	Model	Best value	Worst value	Standard deviation	Best value	Worst value	Standard deviation
Type 2	DRNN-GD	99.7569	98.9138	0.2508	63.2545	63.2516	0.0009
	DRNN-FO	99.3993	99.2397	0.0500	66.1915	66.1902	0.0004
	DLNN	97.7990	97.3510	0.5300	96.5440	95.9341	0.2000
	FRNN-GD	99.4243	90.8717	2.6400	92.2547	92.2313	0.0074
	FRNN-FO	99.5555	98.0625	0.4732	97.1316	97.1276	0.0014
Hypoglycemia	DRNN-GD	99.5629	95.4777	1.2588	88.2813	88.2800	0.0004
	DRNN-FO	99.3006	94.0005	1.7663	94.0005	93.9997	0.0002
	DFNN	98.7660	95.0640	0.5200	90.2760	90.0146	0.7500
	FRNN-GD	99.4631	98.1640	0.1714	90.4692	90.4692	0.0002
	FRNN-FO	99.4397	95.2484	0.5981	97.0173	97.0170	0.0001
Hyperinsulinemia	DRNN-GD	99.8142	98.5615	0.3917	72.3795	72.3724	0.0023
	DRNN-FO	99.8190	98.3940	0.4379	81.3815	81.3771	0.0014
	DFNN	99.3870	85.6420	4.5700	93.7460	84.3910	5.0000
	FRNN-GD	98.7424	98.1640	0.1714	89.1753	89.1530	0.0070
	FRNN-FO	99.2012	95.2484	0.5981	97.1677	95.2269	0.0068
Type 1	DRNN-GD	99.5291	98.4723	0.3089	50.3578	50.3300	0.0088
	DRNN-FO	99.6046	98.7223	0.2778	71.3357	71.3335	0.0007
	DFNN	99.421	96.4370	1.0200	95.6950	94.9120	1.1800
	FRNN-GD	98.6039	85.7233	4.0008	92.1859	92.1767	0.0029
	FRNN-FO	99.6785	94.6001	1.6277	96.0609	96.0501	0.0034

#### Remark

*It is worth noting that the present validation is based on data generated from a pre-validated physiological model of the chaotic diabetes system. This choice arises from the ethical constraints*,* data privacy regulations*,* and limited access associated with obtaining real physiological datasets. The adopted model has been rigorously tested and widely accepted in the literature*,* ensuring that the simulated data reliably reflect real-world Diabetes Mellitus dynamics. While clinical validation would further strengthen the contribution of this work*,* such efforts require institutional approvals and specialized collaborations beyond the current study. Future research will focus on establishing these collaborations to enable experimental and clinical validation of the proposed FRNN-FO framework.*

## Conclusions

This study proposes a Fully Recurrent Neural Network (FRNN) identification framework enhanced by a Fractional-Order (FO) learning algorithm (FRNN-FO) for modeling and identifying the chaotic behavior of insulin-glucose regulatory systems under various diabetes-related disorders. Through extensive simulations involving Type 1 and Type 2 diabetes, hypoglycemia, and hyperinsulinemia, the FRNN-FO model consistently outperformed benchmark approaches, including DRNN-GD, DRNN-FO, DFNN, and FRNN-GD in terms of Root Mean Square Error (RMSE) and FIT percentage (FIT%). By incorporating memory effects via fractional calculus, the model achieved enhanced learning accuracy and stability, effectively capturing the complex, nonlinear dynamics inherent in diabetic conditions. Statistical analysis across multiple trials further validated the robustness and reliability of the FRNN-FO algorithm, exhibiting low error variance and high predictive fidelity. These results underscore the promise of fractional-order neural models in advancing predictive modeling for biomedical applications.

Future work may focus on extending the FRNN-FO framework to the identification of other real-world chaotic systems and on developing advanced recurrent neural architectures with fractional-order learning for improved modeling, prediction, and control of complex dynamical processes.

## Data Availability

The datasets used and/or analysed during the current study available from the corresponding author on reasonable request.
